# LGGC-Net: a local-global graph and color attention-based lightweight CNN for skin cancer classification

**DOI:** 10.1038/s41598-026-48724-8

**Published:** 2026-04-16

**Authors:** Md Aminur Sarker, Md Alamgir Kabir, Md Shakhawat Hossain

**Affiliations:** 1 Department of Computer Science and Engineering, Daffodil International University, Dhaka, Bangladesh; 2 School of Informatics, Kochi University of Technology, Kami, Kochi 782-8502 Japan

**Keywords:** Cancer, Computational biology and bioinformatics, Mathematics and computing

## Abstract

Developing clinically deployable AI systems for skin cancer classification remains challenging due to limited robustness, lack of interpretability and constrained computational resources in hospitals. Although many deep learning models report high accuracy, their large sizes, extensive training requirements and low generalizability hinder practical deployment. In this study, we propose LGGC-Net, a lightweight CNN that incorporates LGGC (Local, Global Graph, and Color) attention to enhance discriminative feature learning while maintaining computational efficiency. Experimental results demonstrate that LGGC attention consistently improves performance across all evaluated CNN backbones. The LGGC-Net model was assessed on external image sets with diverse skin tones to ensure robustness and generalizability under domain shift conditions. Ablation studies analyzed the contribution of individual attention components and explainability was examined using Gradient-weighted Class Activation Mapping++ (Grad-CAM++) and SHapley Additive exPlanations (SHAP). With only 0.81 million parameters, LGGC-Net achieved 88.05% accuracy in 50 epochs, corresponding to 1.761 accuracy per epoch and 108.7 accuracy per million parameters in binary classification. In multiclass settings, it attained 76.1% accuracy on the unseen HAM10000 dataset, with 1.52 accuracy per epoch and 94.0 accuracy per million parameters. In both cases, the area under the curve exceeded 0.93. LGGC-Net consistently outperformed existing methods on deployment-oriented metrics, maintaining stable accuracy. These results indicate that LGGC-Net is an effective, interpretable and potentially deployment-ready solution for practical skin cancer classification.

## Introduction

Skin cancer is one of the most rapidly increasing malignancies worldwide and poses a serious public health challenge. Clinically, diagnosis typically involves visual inspection followed by dermoscopic examination by experienced dermatologists^1^. Prognosis is highly dependent on early detection, particularly for pigmented lesions that may initially appear visually subtle^2^. In dermoscopic practice, skin lesions are commonly categorized into seven major subtypes: melanocytic nevi (NV), benign keratosis-like lesions (BKL), dermatofibroma (DF), vascular lesions (VASC), melanoma (MEL), basal cell carcinoma (BCC), and actinic keratosis (AK)^3^. The first four are generally benign, while MEL and BCC are malignant and AK is considered premalignant. These subtypes differ substantially in biological behavior and clinical risk; for example, melanoma has a high metastatic potential, BCC is locally invasive but rarely metastatic, and AK may progress to squamous cell carcinoma if untreated. Despite these distinctions, accurate subtype differentiation remains challenging due to strong visual overlap, intra-class variability, and inter-class similarity in dermoscopic features^4,5^. Benign lesions may mimic melanoma in terms of pigmentation or asymmetry, while early-stage AK and BCC often exhibit subtle characteristics that are difficult to distinguish. Variations in lesion appearance, anatomical location, and skin tone further complicate diagnosis^6^. Although melanoma represents a smaller fraction of cases, it is the most aggressive subtype and its incidence is rapidly increasing worldwide^7,8^. Consequently, fine-grained multiclass lesion classification is critical for precise diagnosis, appropriate treatment planning, and reducing unnecessary biopsies^9^, highlighting the need for robust automated skin cancer subtyping models.

The diagnostic accuracy of skin cancer subtypes remains highly dependent on clinician expertise and experience, often leading to inter-observer variability and delayed diagnosis. This challenge is further intensified by the rapidly increasing number of patients and the limited availability of specialized dermatologists. Consequently, automated skin cancer classification using artificial intelligence (AI) has emerged as a promising approach to support clinical decision-making by providing fast, objective, and reproducible assessments. AI-driven systems have demonstrated strong potential to improve diagnostic accuracy, enable early-stage detection, reduce clinical workload, and expand access to dermatological care, particularly in resource-constrained settings. However, most existing AI-based approaches rely on computationally intensive training and large, complex models^10^. Although these models often achieve high accuracy in controlled experimental settings, their practical deployment is restricted by large model sizes, high computational demands, and limited computing infrastructure in hospitals and clinical environments^11^. Moreover, many models suffer from limited robustness and poor generalization when applied to real-world clinical data^12,13^. Performance frequently degrades when evaluated on images collected from different hospitals or patient populations due to variations in skin tone, imaging conditions, and data inconsistencies. As a result, despite impressive benchmark performance, the clinical readiness of many AI systems remains questionable. Therefore, the development of robust, lightweight, and generalizable automated skin lesion classification models has become a critical research focus for advancing clinically deployable AI-assisted diagnostic systems.

With the growing demand for lightweight automated skin cancer diagnosis systems, numerous studies have focused on improving both classification performance and computational efficiency. Turker et al.^14^ proposed a lightweight Convolutional Neural Network (CNN) integrating residual and squeeze-and-excitation blocks for binary classification, achieving 91.2% accuracy with 10M parameters over 50 epochs. Huang et al.^15^ introduced an EfficientNet-based lightweight model for seven-class classification on HAM10000, achieving 85.8% accuracy with 17.55M parameters trained for 200 epochs. Dwivedi et al.^16^ proposed a Vision Transformer–based model (LViT) with 86M parameters, reporting 95.82% binary accuracy; however, the model exhibited overfitting and substantial computational complexity despite being described as lightweight, limiting its clinical deployability. Similarly, Ding^17^ and Tang^18^ explored transformer-based lightweight architectures. Baig et al.^2^ presented a ShuffleNet-based lightweight model for seven-class classification on HAM10000 and ISIC 2020, reporting 99.14% accuracy; however, training curves suggest substantially lower effective performance, raising concerns about reliability. Kabir et al.^19^ introduced TinyStudent, a knowledge-distilled model with only 0.35M parameters, achieving 85.45% accuracy, which was further improved to 88.0% using multi-teacher distillation and ensembling (1.05M parameters). Wei et al.^20^ proposed a fine-grained lightweight framework using MobileNet and DenseNet backbones, achieving 85.5% binary accuracy within 50 epochs. More recently, Ozdemir et al.^21^ proposed a hybrid ConvNeXtV2 and separable self-attention model, achieving 93.48% multiclass accuracy with 21.92M parameters on the ISIC 2019 dataset. Asif et al.^22^ introduced SKINC-Net, containing only 0.665M parameters and achieving 98.48% multiclass accuracy on HAM10000. However, this performance was obtained using a dataset-internal 90/10 split without external validation, and AUC values were not reported, limiting assessment of generalization and discriminative robustness in real-world clinical settings.

Most of these methods still rely on large, computationally intensive models; consequently, their practical deployment is often limited, particularly in resource-constrained clinical environments. Some studies have proposed lightweight network architectures; however, model evaluation remains largely restricted to conventional classification metrics, such as accuracy, precision, and recall. As a result, it remains difficult to objectively assess the true deployment feasibility in real-world clinical settings. Evaluating lightweight models using deployment-oriented metrics such as parameter count, model size, computational complexity and inference time is therefore essential for understanding their practical efficiency and enabling meaningful comparison beyond absolute accuracy. A balanced evaluation that jointly considers diagnostic performance and deployment efficiency is crucial for identifying clinically viable solutions. In addition, many existing methods fail to maintain performance when applied to heterogeneous datasets collected from different hospitals. In particular, robustness to skin tone variation and domain shift remains a significant challenge. One contributing factor is that most models are trained primarily on publicly available datasets such as ISIC and HAM10000. Although these datasets are widely used benchmarks, they are predominantly composed of images from light-skinned populations (Fitzpatrick skin types I–II), while darker skin tones (Fitzpatrick skin types III–VI) are substantially underrepresented. Furthermore, explicit skin tone annotations are often unavailable. This demographic imbalance limits the generalizability and clinical fairness of AI models trained on these datasets, especially when deployed in diverse real-world populations. Another common limitation is that many studies train and evaluate models on the same dataset, raising concerns about overfitting and inflated performance estimates. Consequently, external dataset evaluation is necessary to assess robustness under domain shift reliably. Finally, explainability analysis is a critical requirement for clinical deployment, as it provides insight into model decision-making, enhances transparency, and supports clinician trust in AI-assisted diagnostic systems.

To address these challenges, we propose a lightweight CNN framework, LGGC-Net, that leverages the Local–Global Graph Color (LGGC) attention module for accurate, robust, fair, explainable, and deployment-friendly skin cancer classification. The primary contribution of this work is the design of the LGGC attention module and its seamless integration into the LGGC-Net. Although many studies have explored attention mechanisms to enhance feature representation in diverse deep learning based applications, most of them either introduce high computational complexity or lack spatial-context modeling, which motivates the proposed LGGC attention mechanism. Unlike existing approaches, the proposed LGGC attention module jointly captures fine-grained local texture patterns, color-specific lesion characteristics, global contextual information, and inter-region relationships through a unified local, global, graph and color attention mechanism, enabling the network to focus on clinically relevant lesion regions while maintaining low computational complexity. To demonstrate generalizability beyond a single architecture, the proposed LGGC module was also integrated into four widely used CNN backbones: ResNet50, DenseNet121, EfficientNetB0, and MobileNetV2, consistently improving performance across both binary and multiclass skin lesion classification tasks. In contrast to prior studies that emphasize accuracy alone, we evaluated the models using deployment-oriented metrics, including accuracy per epoch, accuracy per model size, accuracy per million parameters, training time, and inference time. LGGC-Net significantly outperformed baseline models and existing AI-based methods in deployment compatibility while maintaining sufficient diagnostic performance. Robustness and generalization were further evaluated under realistic domain-shift conditions by training on ISIC and testing on the partially heterogeneous HAM10000 dataset in both binary and multiclass settings. In addition, to explicitly address skin tone bias and adaptability, we evaluated the model on a diverse skin tone dataset (DST-50) comprising images from Fitzpatrick skin types III–VI. Explainability analyses using Gradient-weighted Class Activation Mapping++ (Grad-CAM++)^23^ and SHapley Additive exPlanations (SHAP)^24^ demonstrate that LGGC-Net consistently attends to diagnostically meaningful regions, supporting transparency and clinical trust. In summary, the main contributions of this work are threefold:

1. The development of the LGGC attention module, which integrates local, global, graph-based, and color-aware feature interactions for efficient representation learning.
2. Its integration into a lightweight CNN architecture (LGGC-Net) and several standard backbones to evaluate its architectural generalizability.
3. A comprehensive evaluation including deployment-oriented efficiency metrics and robustness analysis across datasets with varying skin tone distributions.

Overall, LGGC-Net provides a robust, efficient, and clinically deployable solution for real-world skin cancer screening.

## Related works

Automated skin cancer classification has been widely investigated using deep learning, motivated by the need for accurate and scalable diagnostic support systems. Despite substantial progress, existing approaches differ widely in terms of computational efficiency, robustness, fairness, and deployment ability. In this section, we review prior studies with a focus on CNN-based lightweight models, transformer-based lightweight models, attention-based models, and research addressing robustness, bias, and explainability in skin lesion classification.

### CNN-based lightweight models

CNNs remain the dominant paradigm for skin lesion classification due to their strong performance and relative training stability. To improve computational efficiency, several studies have proposed lightweight CNN architectures or reduced-capacity variants of standard backbones. Models based on ResNet, EfficientNet, MobileNet, DenseNet and Shuffle-Net have demonstrated competitive classification performance with reduced parameter counts^2,15,20,25–29^. However, in many cases, performance gains are achieved through extended training schedules or extensive data augmentation, which may reduce practical efficiency. Knowledge distillation and model compression have also been explored to further reduce model size. Kabir et al.^19^ introduced TinyStudent, achieving reasonable accuracy with a substantially reduced number of parameters. While such approaches are promising for memory-constrained environments, their performance generally remains below that of larger teacher models and ensemble-based improvements increase architectural complexity. Moreover, evaluation is often limited to a single dataset, making it difficult to assess robustness across different data distributions. More recent architectures, such as SKINC-Net^22^, report high accuracy with very small parameter counts. However, these results are commonly obtained using random train–test splits from the same dataset, without external validation. In addition, several studies report only accuracy-based metrics, limiting insight into discriminative performance across decision thresholds. Collectively, while CNN-based lightweight models reduce computational cost, their deployment readiness remains difficult to assess due to limited evaluation beyond standard classification metrics.

### Transformer-based lightweight models

Transformer-based models have been introduced to capture long-range dependencies and global contextual information in dermoscopic images. Vision Transformer (ViT) variants such as LViT^16^, HI-MViT^17^, and SkinSwinViT^18^ have reported improved performance for visually similar lesion categories. However, despite being described as lightweight, many of these models contain a large number of parameters, resulting in increased computational demands that may limit their applicability in resource-constrained clinical settings. Hybrid architectures combining CNN backbones with attention mechanisms have been proposed to balance global context modeling and efficiency. Methods incorporating multi-scale attention^30^, separable self-attention^21^, or lightweight attention modules^31,32^ demonstrate improved feature representation. Nevertheless, these approaches often introduce additional architectural complexity and rely on dataset-specific preprocessing strategies. Furthermore, transformer based models are typically evaluated using standard performance metrics, with limited reporting of inference latency or deployment-oriented efficiency.

### Attention-based models

Attention mechanisms have emerged as a powerful approach for improving deep learning-based skin lesion classification by enabling models to focus on diagnostically relevant regions while suppressing background noise. Early studies demonstrated that integrating attention into CNNs enhances feature discrimination. For instance, soft-attention mechanisms have been shown to consistently improve classification accuracy across multiple backbone architectures by emphasizing salient lesion features^33^. Similarly, residual attention learning frameworks^34^ exploit hierarchical feature interactions to generate attention maps without introducing excessive computational burden. Building on these foundations, several works explored channel and spatial attention mechanisms to refine feature representations. Dual attention strategies such as EDA-ResNet50^35^ combine channel and spatial recalibration with multi-scale feature extraction to capture subtle lesion variations. Channel-focused designs, including Eff2Net^36^ and multi-scale channel attention models^37^, aim to improve efficiency while reducing parameter complexity. In parallel, lightweight architectures such as attention-enhanced MobileNet^38^ highlight the importance of designing deployable solutions for clinical environments.

A major line of studies investigated multi-scale and hierarchical attention modeling, where lesion characteristics are captured at different resolutions. Representative approaches include MuRANet^39^, GMAB-based CNNs^40^, MSLANet^41^, and EFAM-Net^42^, all of which leverage multi-scale attention blocks and feature fusion strategies to improve classification performance. While effective, these methods often rely on stacking multiple attention modules or complex fusion pipelines, leading to increased computational overhead. Another important direction focuses on global context modeling and hybrid attention architectures. Methods such as Skin-GAB^43^ encode spatial and channel dependencies jointly, while attention-integrated transfer learning frameworks^44^ explore different attention types to enhance feature learning. More recent approaches, including DSCATNet^45^ and RCSABC-Conformer^46^, incorporate transformer-based self-attention to capture long-range dependencies. Although these models demonstrate strong performance, they typically introduce substantial computational complexity and are less suitable for lightweight deployment.

To further improve robustness, several studies integrate attention with cost-sensitive learning and ensemble strategies. For example, attention-guided cost-sensitive frameworks^47^ and multi-modal attention models^48^ enhance performance under class imbalance and complex feature distributions. Similarly, Att2ResNet^49^ investigates optimal attention placement within deep architectures. However, these approaches often involve multi-stage pipelines or ensemble learning, reducing interpretability and increasing system complexity. Moreover, comparative analyses^50^ suggest that attention mechanisms do not always yield consistent improvements across different datasets and architectures, highlighting the need for more principled and efficient designs.

Despite these advancements, existing attention-based models generally exhibit a fragmented design philosophy, where local feature refinement (e.g., channel or spatial attention) and global dependency modeling (e.g., self-attention or transformers) are addressed separately. Additionally, many approaches incur high computational cost, rely on complex multi-branch or ensemble frameworks, and often overlook color-specific cues, which are crucial for skin lesion classification. These limitations restrict their applicability in real-world, resource-constrained clinical settings.

### Robustness, bias and explainability-oriented studies

Recent studies have highlighted the importance of robustness and fairness in automated skin lesion analysis. Variations in skin tone, image acquisition conditions and dataset composition have been shown to influence model performance^51,52^. Approaches addressing skin tone bias and demographic disparities have demonstrated improved consistency across patient groups, although they often require additional training objectives or specialized preprocessing. Other works have examined robustness to visual bias, preprocessing variation, and domain shift^25,27^. While these strategies can improve generalization, they frequently rely on complex augmentation pipelines or ensemble-based designs, increasing system complexity. Methods targeting label noise and heterogeneous data sources further improve reliability^53,54^, but typically at the cost of increased computational overhead. Explainability has received growing attention as a prerequisite for clinical adoption. However, many lightweight and transformer-based models provide limited analysis of model attention or decision rationale, which may restrict clinical interpretability and trust^2,55^.

### Summary and motivation

CNN-based, transformer-based, and attention-augmented models have advanced skin lesion classification, yet each has limitations. Lightweight CNNs reduce parameters but often lack global context and interpretability. Transformers capture long-range dependencies but incur high computational cost. Attention-based approaches improve focus on salient regions, yet fragmented designs, multi-stage pipelines, and the absence of color-aware modeling reduce efficiency and applicability. While existing studies demonstrate that CNNs, transformers and attention based models can achieve strong classification performance, challenges remain in deployment-oriented evaluation, robustness across heterogeneous datasets and skin tones, computational efficiency, and interpretability. These limitations motivate the development of models that jointly address accuracy, efficiency, robustness, fairness, and explainability, providing a foundation for clinically deployable skin cancer screening systems.

## Materials and methodology

### Ethics statement

The datasets employed in this study comprises anonymized human skin images that are publicly available on (ISIC Archive). As all personal identifiers were removed and the data were fully de-identified, the university research ethics committee waived the requirement for Institutional Review Board (IRB) approval. This study does not involve direct interaction with human participants or access to identifiable private information, thereby adhering to established ethical standards for human subjects research.

### Dataset

In this study, dermoscopic images were collected from the ISIC Archive^56^, one of the largest publicly available repositories for skin cancer imaging. For the binary classification experiments, we randomly selected 10,000 benign and 10,000 malignant images, which are collectively referred to as the SKIN2025 dataset in this study. The HAM10000^3^ and ISIC 2019^57^ datasets also belong to the ISIC archive. We ensured that the HAM10000^3^ and ISIC 2019^57^ images are not included in the SKIN2025. The benign class includes NV, BKL, DF, and VASC. The malignant class includes MEL, BCC and AK subtypes. A small number of SCC images were also present in the malignant set. As SCC commonly develops from AK, these images were grouped with the AK category and included in the malignant class. Notably, invasive SCC images are not explicitly labeled as a separate class in the ISIC datasets, and SCC cases are primarily represented through AK-related categories. In contrast, such SCC cases are absent from the AK class of the HAM10000 dataset^3^. The SKIN2025 dataset was partitioned into 60% for training, 20% for validation and 20% for testing the binary classification models. For multiclass classification, we used the ISIC 2019 dataset^57^, which comprises 24,703 dermoscopic images spanning seven lesion subtypes. The dataset contained 12,875 NV images (52.11%), followed by MEL with 4,522 images (18.31%), BCC with 3,323 images (13.45%), and BKL with 2,624 images (10.62%). Minority classes, including AK, DF and VASC, together account for less than 6.5% of the dataset. The dataset was divided into 17,301 images (70.0%) for training, 3,696 images (15.0%) for validation and 3,706 images (15.0%) for testing. Although this class imbalance reflects real-world clinical prevalence, it poses challenges for deep learning models. Therefore, robust and class-sensitive evaluation metrics were adopted in this study to ensure reliable performance assessment.

To assess the robustness and generalization capability of the proposed models, an external evaluation was conducted using the HAM10000 dataset, another widely used benchmark in dermoscopic image analysis. For binary classification, 8,000 benign and 2,000 malignant images were selected. For multiclass evaluation, 10,015 images covering the same seven lesion subtypes were extracted. These samples were not used during training or validation, enabling a rigorous assessment of model performance under partially heterogeneous data conditions. However, the HAM10000 and ISIC images represent the same patient population and similar skin tones (Fitzpatrick skin types I–II). Therefore, we prepared a dataset of 50 images with diverse skin tones (DST-50), comprising 50 malignant and 50 benign images, collected from different patient populations with varying skin tones (Fitzpatrick skin types III–VI). This dataset was used to validate the binary classification model. The data distributions for training and testing the binary and multiclass models are presented in Tables 1 and 2, respectively.

**Fig. 1:**
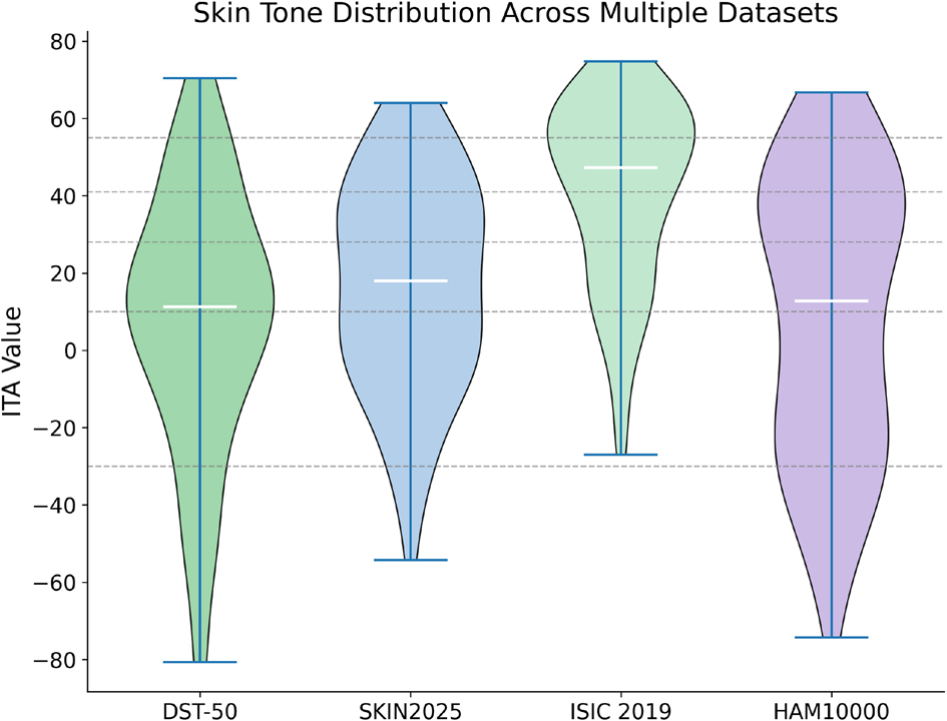
Skin tone distribution in the datasets.

Further, we plotted the distribution of skin tones across the datasets, as shown in Fig. 1. The violin plot summarizes skin tone variations using the Individual Typology Angle (ITA) score, computed after extracting skin regions and converting images to the CIE Lab color space. ITA is an objective measure of skin pigmentation, where higher values indicate lighter skin tones and lower values indicate darker skin tones. The continuous ITA scores were mapped to clinically recognized Fitzpatrick skin types, with ITA $\ge 41$ corresponding to Types I–II, ITA between 10 and 40 to Types III–IV, and ITA $\le -30$ to Types V–VI. The width of each violin represents the density of samples at a given ITA range, while the central marker indicates the median. This visualization facilitates direct comparison of skin tone diversity and highlights potential imbalances in Fitzpatrick skin type representation across datasets. This plot clearly indicates that the DST-50 is more diverse in skin tones.

**Table 1:** Distribution of binary skin lesion classes across training, validation, testing, and external evaluation datasets.

Dataset	SKIN2025			HAM10000^3^	DST-50
Class	Train	Validation	Test	External test	External test
Benign	6000	2000	2000	8000	50
Malignant	6000	2000	2000	2000	50
Total	12,000	4000	4000	10,000	100

**Table 2:** Distribution of skin lesion subtypes across training, validation, testing and external evaluation datasets.

Dataset	ISIC 2019^57^			HAM10000^3^
Class	Train	Validation	Test	External test
AK	607	130	130	327
BCC	2327	497	499	514
BKL	1838	392	394	1099
DF	167	36	36	115
MEL	3167	677	678	1113
NV	9018	1926	1931	6705
VASC	177	38	38	142
Total	17,301	3696	3706	10,015

### Deployment-oriented evaluation metrics

To evaluate deployment compatibility, conventional accuracy-based metrics alone are insufficient. Real-world clinical deployment requires models that are accurate, efficient, balanced, and fast under resource constraints. Therefore, we adopt deployment-oriented metrics that jointly assess diagnostic reliability, computational efficiency, model compactness, and training practicality.

#### Performance balance and stability metrics

We first assess classification balance and reliability, which are essential for clinical safety and fairness. The Stability Index (SI) measures the balance between sensitivity and specificity. A high SI value indicates consistent performance across positive and negative classes, reducing bias toward either benign or malignant predictions. Such balance is critical for minimizing both false negatives and false positives in clinical screening.

1 $$\begin{aligned} \begin{aligned} \text {Difference}&= |\text {Sensitivity} - \text {Specificity} |\\ \text {Stability Index (SI)}&= 1 - |\text {Sensitivity} - \text {Specificity} |\end{aligned} \end{aligned}$$

The F1 Difference Ratio further evaluates class-wise balance by quantifying the disparity between benign and malignant F1-scores. Higher values reflect more equitable class-level performance, which is particularly important in imbalanced datasets and for maintaining clinical trust.

2 $$\begin{aligned} \text {F1 Difference Ratio} = 1 - \left| \text {F1}_{\text {Benign}} - \text {F1}_{\text {Malignant}} \right| \end{aligned}$$

#### Efficiency-oriented deployment metrics

We next examine computational efficiency, a primary factor influencing real-world deployability. The Inference Efficiency metric measures how much diagnostic accuracy is achieved per unit of inference time. Models with high inference efficiency are better suited for real-time screening, point-of-care applications, and high-throughput clinical workflows.

3 $$\begin{aligned} \text {Inference Efficiency} = \frac{\text {Accuracy}}{\text {Inference Time (s)}} \end{aligned}$$

The Training Efficiency metric evaluates how effectively a model translates training time into predictive accuracy. This metric is especially relevant for model retraining, adaptation to new datasets, and deployment in environments with limited computational resources.

4 $$\begin{aligned} \text {Training Efficiency} = \frac{\text {Accuracy}}{\text {Training Time (s)}} \end{aligned}$$

#### Joint performance–efficiency evaluation

To assess the balance between accuracy and computational cost, we employ the Efficiency-Performance Ratio (EPR). This metric jointly considers model accuracy, inference time, and model size, enabling direct comparison of models in terms of their overall deployment feasibility. Higher EPR values indicate models that achieve strong diagnostic performance while maintaining low computational and memory overhead.

5 $$\begin{aligned} \begin{aligned} \text {Efficiency-Performance Ratio} = {} \frac{\text {Accuracy \%}}{\text {Model Size (MB)} \times \text {Inference Time (s)}} \end{aligned} \end{aligned}$$

#### Model compactness and resource utilization metrics

To explicitly measure how efficiently models use parameters and memory, we include Accuracy per Million Parameters, which highlights architectures that deliver higher predictive performance with fewer parameters, favoring lightweight and memory-efficient designs. Accuracy per Model Size evaluates performance relative to storage requirements, a critical consideration for edge devices and embedded clinical systems.

6 $$\begin{aligned} & \begin{aligned} \text {Accuracy per Million Parameters} {}= \frac{\text {Accuracy \%}}{\text {Total Parameters (in millions)}} \end{aligned} \end{aligned}$$

7 $$\begin{aligned} & \text {Accuracy per Model Size} = \frac{\text {Accuracy \%}}{\text {Model Size (MB)}} \end{aligned}$$

#### Convergence and training practicality metric

Finally, Accuracy per Epoch is used to assess learning efficiency and convergence speed. Models that achieve competitive accuracy in fewer epochs reduce training cost and energy consumption, making them more practical for iterative development and deployment under constrained training budgets.

8 $$\begin{aligned} \text {Accuracy per Epoch} = \frac{\text {Accuracy \%}}{\text {Number of Epochs}} \end{aligned}$$

We also report other commonly used computational efficiency measures. GFLOPs (Giga Floating Point Operations) quantify the total number of floating-point operations required for a single forward pass, reflecting the model’s computational complexity. GMACs (Giga Multiply Accumulate Operations) represent the number of multiply–accumulate operations performed during inference and provide another estimate of computational workload. Latency (ms) measures the time required to process a single input image during inference and indicates the model’s responsiveness. Throughput (images/s) represents the number of images processed per second and reflects suitability for high-volume clinical screening. Peak GPU memory (MB) reports the maximum memory usage during inference, indicating the model’s memory efficiency and practical deployability on resource-constrained hardware. Together, these metrics provide a comprehensive, deployment-centric evaluation framework that captures not only diagnostic accuracy but also efficiency, fairness, stability, scalability, and real-world usability, enabling objective comparison and selection of models suitable for clinical deployment.

### Proposed LGGC-net

The proposed LGGC-Net is a light-weight CNN for skin cancer classification. The network passes a 224$\times$224$\times$3 input image across four successive blocks. Conventional Conv2D layers with Batch Normalization (BN) and Leaky ReLU activations are used in each block to stabilize learning and enhance feature learning. Spatial information is efficiently encoded using depthwise convolution, and the proposed LGGC attention module fine-tunes feature representations by selecting crucial local, color, graph, and global information. The feature size is compressed, and relevant features are emphasized using Max Pooling. In the final block, a Conv2D layer with 512 filters is used, with BN, Leaky ReLU, and depthwise convolution for feature extraction at higher levels of the input. The classification head includes Global Average Pooling (GAP) to compress feature maps and two fully connected (Dense) layers with Leaky ReLU and Dropout to prevent overfitting. Subsequently, a Dense layer with sigmoid activation outputs the class probability for each. With such a structure, LGGC-Net effectively learns and integrates local and global feature information to accurately classify skin lesions.

**Fig. 2:**
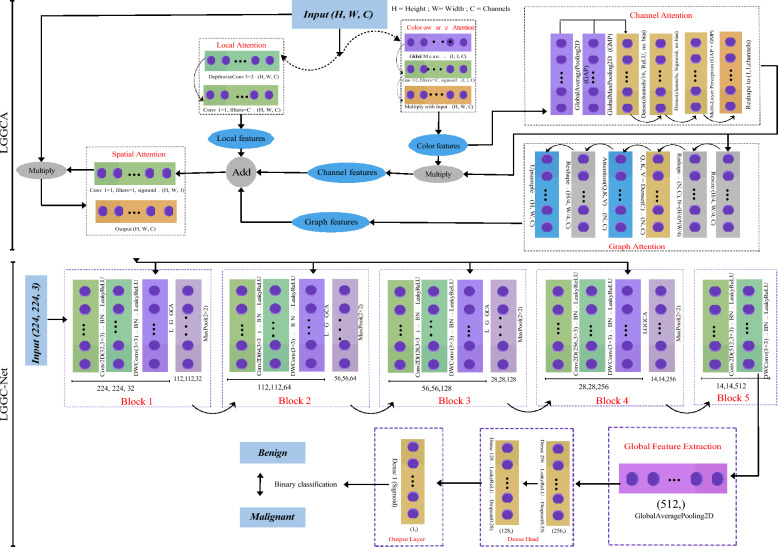
Architecture of proposed LGGC-Net model with LGGC attention module.

Figure 2 describes that the proposed network has two main parts: The top part represents the LGGC attention (LGGCA) module, and the bottom part represents our proposed LGGC-Net model. The step-wise algorithm of the LGGC-Net in Algorithm 1. The whole process starts with the input skin image (224, 224, 3). First this image is passed into a special LGGC attention module which looks at the image in a few different ways: one part focuses on small local regions (using a small filter and a 1$\times$1 filter) to get local features, another part computes a spatial attention map that highlights important areas in the image, and color-aware part averages the colors across the image to adjust the strength of each color channel. There is also a channel attention path that uses global pooling to identify which of the many feature channels is most important, and a graph attention path that treats the image features as a graph to capture relationships between distant parts of the image. The local, channel, and graph attentions are combined, and the resulting feature map is then multiplied by the spatial attention map to focus on important regions. This produces an output with the same height, width, and channels as the input image, and now these features are weighted by all the attentions. Next, this weighted output is fed into the main part of LGGC-Net, which consists of 5 sequential layers. In Block 1, the image (224$\times$224$\times$3) passes through a 3$\times$3 convolution with 32 filters and a LeakyReLU activation, then another 3$\times$3 convolution with 32 filters and the same activation. Then the LGGC module is applied again, and finally, a max-pooling reduces the image size to 112$\times$112 while keeping 32 channels. In Block 2, the 112$\times$112$\times$32 data goes through two more 3$\times$3 convolutions (each with 64 filters) and LeakyReLU activation, then two more 3$\times$3 convolutions (still 64 filters) and the same activation, then the LGGC module again, and a max-pooling to get 56$\times$56$\times$64. Block 3 does a similar thing with three 3$\times$3 convolutions (with 128 filters) and LeakyReLU activation, then three more 3$\times$3 convolutions (still 128 filters) and the same activation, followed by LGGCA and pooling to 28$\times$28$\times$128. Block 4 again has three 3$\times$3 convolutions (with 256 filters) and LeakyReLU activation, then three more 3$\times$3 convolutions (still 256 filters) and the same activation, followed by LGGC and pooling to 14$\times$14$\times$256. Block 5 has three 3$\times$3 convolutions (with 512 filters) and activations, then three more 3$\times$3 convolutions (also with 512 filters) and activations, followed by the LGGC module, producing 14$\times$14$\times$512 without extra pooling. Now we have 14$\times$14$\times$512 features describing the image, and we apply global average pooling to reduce each 14$\times$14 feature map to a single value, yielding 512 values in total. These 512 numbers are then passed through a small dense (fully-connected) head: first to 256 neurons with LeakyReLU, then to 128 neurons with LeakyReLU, and finally to a single output neuron. The last output neuron uses a sigmoid activation function to produce a value between 0 and 1, corresponding to the probability that the image is malignant or benign.

### LGGC attention module

The proposed LGGC attention block is designed to enhance feature representation by integrating multiple complementary attention mechanisms in a unified and computationally efficient manner. The step-wise algorithm of the attention module is given in Algorithm 2. The block begins with a local attention module implemented using depthwise and pointwise convolutions to capture fine-grained spatial details and local texture patterns, which are critical for distinguishing subtle lesion characteristics. Then, a color-aware attention module is introduced to emphasize discriminative color information, which is vital for differentiating malignant and benign skin lesions. This module selectively amplifies informative color responses, enabling the network to better exploit chromatic variations commonly observed in dermoscopic images. Subsequently, a channel attention module is applied, which uses both global average pooling (GAP) and global max pooling (GMP) to model global channel-level statistics. By learning adaptive channel-wise weights, this step allows the network to prioritize the most informative feature channels while suppressing less relevant ones.

To capture long-range dependencies and global contextual relationships, a graph-based attention mechanism is incorporated. In this module, the feature map is treated as a set of nodes, and pairwise relationships between spatial locations are modeled using a lightweight self-attention formulation.

9 $$\begin{aligned} \textrm{Attn}(Q,K,V) = \textrm{Softmax}\left( \frac{QK^{T}}{\sqrt{C}}\right) V, \end{aligned}$$

which enables effective global interaction without requiring an explicit adjacency matrix. This design significantly reduces computational overhead while still capturing meaningful long-distance correlations.

The outputs of the local, color-aware, channel, and graph attention modules are then fused element-wise, followed by a spatial attention module that generates an attention map highlighting diagnostically relevant regions. This final refinement step ensures that the network focuses on spatially important lesion areas while suppressing background noise. Overall, the LGGC attention module facilitates hierarchical feature refinement by progressively integrating local texture information, color-specific cues, global channel importance and long-range relational context. The module was implemented using TensorFlow and Keras, leveraging depthwise convolutions for efficient local feature extraction, pooling operations for channel and color refinement and a lightweight global attention mechanism for relational modeling.

In essence, the LGGC attention module is structured to learn image features in a *coarse-to-fine* and *local-to-global* manner. Local attention captures fine textures, color-aware attention refines chromatic patterns, channel attention identifies globally critical feature responses and graph attention models long-range spatial dependencies. Finally, spatial attention consolidates these representations by emphasizing clinically meaningful regions. This sequential design enables the network to develop a comprehensive and balanced understanding of skin lesion characteristics, thereby improving classification performance while maintaining computational efficiency.

**Algorithm 1:**
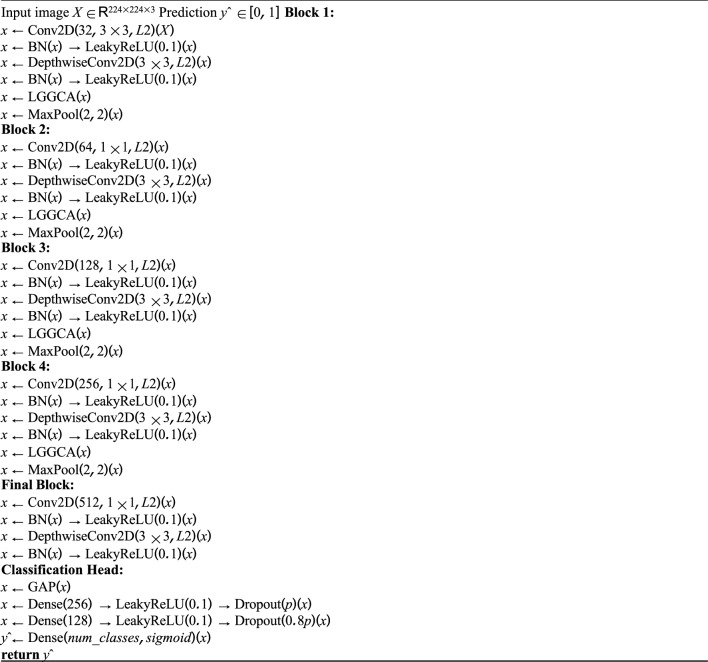
LGGC-Net $$(X, num\_classes=1, \lambda =10^{-5}, p=0.35)$$

**Algorithm 2:**
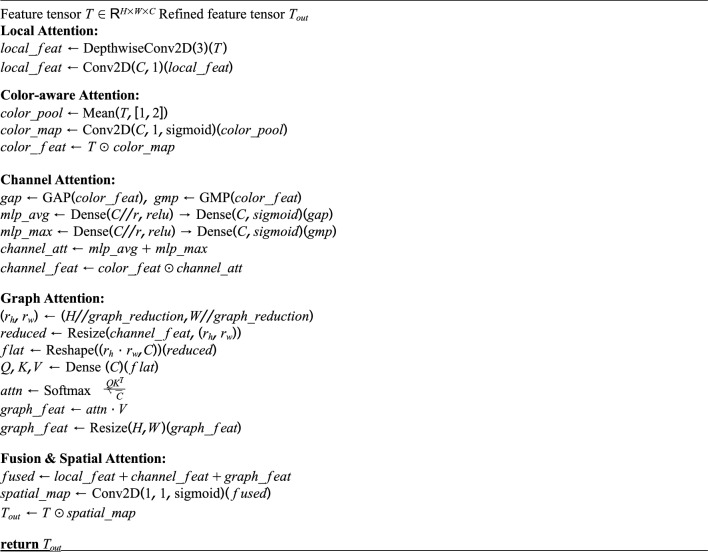
LGGC attention module $(T, reduction=16, graph\_reduction=4)$

#### Mathematical formulation

Let the input feature map be denoted as

$$X \in \mathbb {R}^{H \times W \times C},$$

where *H*, *W*, and *C* represent the height, width, and number of channels, respectively.

*(1) Local attention* To extract fine-grained local texture information, a depthwise convolution followed by a pointwise ($1\times 1$) convolution is applied:

$$F_L = \text {Conv}_{1\times 1}\big (\text {DWConv}_{3\times 3}(X)\big ),$$

where $F_L \in \mathbb {R}^{H \times W \times C}$ denotes the local feature response. This operation preserves spatial resolution while efficiently capturing neighborhood-level patterns.

*(2) Color-aware attention* To emphasize discriminative color characteristics, global spatial averaging is first applied to the input feature map:

$$v_c = \operatorname {Mean}_{(H,W)}(X) \in \mathbb {R}^{1 \times 1 \times C}.$$

A channel-wise color attention vector is then obtained using a $1\times 1$ convolution followed by a sigmoid activation:

$$M_c = \sigma (W_c * v_c),$$

where $W_c$ denotes the learnable convolution kernel and $\sigma (\cdot )$ is the sigmoid function. The color-refined feature map is computed as

$$F_C = X \odot M_c,$$

where $\odot$ represents element-wise multiplication.

*(3) Channel (Global) attention* To model global inter-channel dependencies, both Global Average Pooling (GAP) and Global Max Pooling (GMP) are applied to $F_C$:

$$g_{\text {avg}} = \text {GAP}(F_C), \qquad g_{\text {max}} = \text {GMP}(F_C).$$

The pooled descriptors are passed through a shared two-layer multilayer perceptron (MLP):

$$s_{\text {avg}} = \sigma \!\left( W_2\,\delta (W_1 g_{\text {avg}})\right) , \qquad s_{\text {max}} = \sigma \!\left( W_2\,\delta (W_1 g_{\text {max}})\right) ,$$

where $\delta (\cdot )$ denotes the ReLU activation, $W_1 \in \mathbb {R}^{C \times (C/r)}$ and $W_2 \in \mathbb {R}^{(C/r) \times C}$ are learnable weights, and *r* is the channel reduction ratio. The final channel attention map and refined feature representation are given by

$$M_{ch} = s_{\text {avg}} + s_{\text {max}}, \qquad F_G = F_C \odot M_{ch}.$$

*(4) Graph attention (local–global context modeling)* To capture long-range spatial relationships, the feature map $F_G$ is resized to a reduced spatial resolution $(r_h, r_w)$ and reshaped into a sequence:

$$Z = \text {Reshape}\big (\text {Resize}(F_G)\big ) \in \mathbb {R}^{N \times C}, \quad N = r_h \times r_w.$$

Query, Key, and Value matrices are obtained via linear projections:

$$Q = ZW_Q, \qquad K = ZW_K, \qquad V = ZW_V,$$

where $W_Q, W_K, W_V \in \mathbb {R}^{C \times C}$. The attention matrix and aggregated graph features are computed as

$$A = \text {Softmax}\!\left( \frac{QK^\top }{\sqrt{C}}\right) , \qquad F_{\text {graph}} = AV.$$

The output is then reshaped and upsampled to the original spatial dimensions:

$$F_{Gg} = \text {Resize}\!\left( \text {Reshape}(F_{\text {graph}}, [r_h, r_w, C])\right) .$$

*(5) Feature fusion and spatial attention* The outputs from the local, channel, and graph attention modules are fused through element-wise summation:

$$F_{\text {fusion}} = F_L + F_G + F_{Gg}.$$

To further emphasize spatially important lesion regions, a spatial attention map is generated by applying a $1\times 1$ convolution followed by a sigmoid activation:

$$M_s = \sigma (W_s * F_{\text {fusion}}),$$

where $W_s$ denotes the learnable convolution kernel. Finally, the output of the LGGC module is obtained by reweighting the original input feature map using the spatial attention map:

$$Y = X \odot M_s,$$

where $*$ represents the convolution operation and $\odot$ denotes element-wise multiplication.

Through this fusion and spatial refinement process, the LGGC attention module integrates local texture information, color-aware cues, global channel dependencies, and long-range contextual relationships, enabling the network to selectively focus on diagnostically relevant regions of skin lesions while maintaining computational efficiency.

#### Theoretical justification of complexity

The proposed LGGC attention module is designed to enhance feature discrimination while maintaining computational efficiency. It integrates four complementary attention mechanisms, namely *Local*, *Color-aware*, *Channel (global)* and *Graph* attention, each capturing distinct yet synergistic characteristics of skin lesion images. Through lightweight operations and dimensionality reduction strategies, the LGGC module effectively models both fine-grained local patterns and long-range contextual dependencies with limited computational overhead.

*Local attention* The local attention branch captures fine spatial details and lesion boundaries using depthwise and pointwise convolutions. Depthwise separable convolution significantly reduces computation compared to standard convolution while preserving spatial sensitivity. The resulting complexity is linear with respect to the input dimensions:

10 $$\begin{aligned} \mathscr {O}_{\text {local}} = \mathscr {O}(HWC), \end{aligned}$$

where *H*, *W*, and *C* denote the height, width, and number of channels of the feature map.

*Color-aware attention* The color-aware attention module highlights discriminative chromatic cues by computing channel-wise global statistics followed by a $1\times 1$ convolution and sigmoid activation. This operation emphasizes lesion color contrast while introducing minimal overhead:

11 $$\begin{aligned} \mathscr {O}_{\text {color}} = \mathscr {O}(C^2). \end{aligned}$$

*Channel attention* The channel attention module recalibrates feature importance using global average pooling (GAP) and global max pooling (GMP), followed by a shared two-layer MLP with reduction ratio *r*. This mechanism enhances informative channels while suppressing redundant ones, with complexity:

12 $$\begin{aligned} \mathscr {O}_{\text {channel}} = \mathscr {O}\left( \frac{C^2}{r}\right) . \end{aligned}$$

*Graph attention* To capture long-range spatial dependencies, graph attention is applied to a spatially reduced feature map. The input feature map is downsampled by a factor $r_g$, yielding $N = \frac{HW}{r_g^2}$ nodes. Self-attention is then computed over these nodes, resulting in:

13 $$\begin{aligned} \mathscr {O}_{\text {graph}} = \mathscr {O}(N^2 C). \end{aligned}$$

The reduction factor $r_g > 1$ significantly lowers computational cost while preserving global contextual information.

*Fusion and spatial attention* The outputs of the local, channel, and graph attention branches are fused via element-wise summation and refined using a spatial attention map generated by a $1\times 1$ convolution followed by a sigmoid activation. This step guides the network to focus on diagnostically relevant lesion regions, with complexity approximately:

14 $$\begin{aligned} \mathscr {O}_{\text {fusion}} = \mathscr {O}(HWC). \end{aligned}$$

*Overall complexity* Combining all components, the total computational complexity of the LGGC attention module is given by:

15 $$\begin{aligned} \mathscr {O}_{\text {LGGC}} = \mathscr {O}(HWC) + \mathscr {O}(C^2) + \mathscr {O}\left( \frac{C^2}{r}\right) + \mathscr {O}(N^2 C), \end{aligned}$$

where the reduction factors *r* and $r_g$ ensure scalability and efficiency.

Overall, the LGGC attention module provides a principled balance between representational richness and computational efficiency. By jointly modeling local textures, color cues, global channel dependencies, and long-range spatial relationships within a single lightweight module, it enables accurate and robust skin lesion classification while remaining suitable for deployment in real-world clinical environments.

## Training strategy

In the offline learning environment, the model is trained on the entire dataset in mini-batches using the standard Keras fit() function. All images are resized to $224 \times 224$, with an input shape of (224, 224, 3). The model is trained with a batch size of 64 for 50 epochs. To enhance data diversity and reduce overfitting, data augmentation techniques are applied, including random rotation ($\pm 10^\circ$), width and height shifts (0.1), and zoom (0.1). Both training and validation data generators are employed during training. To further improve generalization and stabilize optimization, multiple callbacks are utilized, including EarlyStopping, ReduceLROnPlateau, and ModelCheckpoint. These callbacks help prevent overfitting and dynamically adjust the learning rate based on validation performance. The model is optimized using the binary cross-entropy loss function and the Adam optimizer with a learning rate of $1 \times 10^{-4}$. Model performance is evaluated using accuracy and the area under the ROC curve (AUC) as evaluation metrics.

On the other hand, the online learning environment trains the model incrementally, updating the network weights after each mini-batch (batch size = 64) rather than training on the entire dataset at once. The input image resolution and batch size were fixed at $224 \times 224$ and 64, respectively, and the model was trained for 15 epochs. All images were normalized using a rescaling factor of 1/255. Data augmentation included random rotation (10$^\circ$), width and height shifts (0.1), zoom (0.1), and nearest-neighbor filling mode. The proposed LGGC-Net, a customized CNN architecture, incorporates the LGGC attention module, which integrates local, channel, color, and graph attention mechanisms to enhance feature extraction. Training was implemented manually using a for-loop with the train_on_batch() function, allowing the model to process each batch independently from the training generator. Validation was performed after each epoch using the evaluate() function. The learning rate was reduced by 5% after every epoch, and early stopping was implemented manually based on the validation loss. After training, the model was evaluated on the test dataset to measure accuracy, loss, and AUC, and to generate evaluation metrics such as ROC curves. In addition, Grad-CAM++ and SHAP visualizations were employed to highlight the most influential regions contributing to the model’s predictions.

In contrast, offline learning trains the model on the entire dataset across epochs using the standard fit() function, without updating weights on a batch-by-batch basis. Online learning, implemented via train_on_batch(), enables finer control over the learning process, including learning rate scheduling, memory usage, and adaptability during training. For a fair comparison, MobileNetV2, EfficientNetB0, DenseNet121, and ResNet50 were trained following the same offline learning strategy as LGGC-Net. Images were preprocessed using preprocess_input() for the pretrained models, while LGGC-Net used rescaling with 1/255, ensuring consistency with each model’s original training configuration. Pretrained weights were kept frozen, and the LGGC attention module was appended, followed by global average pooling and a final fully connected layer. A softmax activation was used for multi-class classification, whereas a sigmoid activation was employed for binary classification. The batch size, learning rate, and optimizer were kept constant across all models to ensure fair training conditions. All experiments were conducted on Kaggle using two NVIDIA T4 GPUs. GPU utilization varied due to the lightweight nature of LGGC-Net and occasional CPU-based data loading. This observation demonstrates that LGGC-Net can be trained efficiently without requiring full GPU resources, making it suitable for deployment in low-resource environments.

### Reproducibility statement

To ensure reproducibility, all experiments were conducted with a fixed seed. For primary binary, multiclass and attention variant based ablation study a seed of 42 was used across all models. For controlled binary classification experiments evaluating the LGGC attention mechanism, multiple CNN architectures were trained using seeds 123, 2024 and 999, with results reported as mean ± standard deviation to capture variability from random initialization and training stochasticity. Dataset splits are detailed in Tables 1 and 2, with the training, validation and test partitions applied consistently across all experiments. The training protocol, including data preprocessing, augmentation and evaluation procedures, was standardized to enable reliable reproduction of our results.

## Results

This section presents a comprehensive evaluation of the proposed LGGC attention module and its impact on skin cancer classification performance and deployment feasibility. First, the effectiveness of LGGC attention was assessed on standard CNN backbones such as ResNet50, MobileNetV2, DenseNet121, and EfficientNetB0, as well as a custom-made CNN, LGGC-Net, under both binary and multiclass skin cancer classification settings using conventional performance metrics. Next, an ablation study was conducted to quantify the individual contributions of each attention component within the LGGC module. To assess real-world applicability, deployment compatibility was evaluated using deployment-oriented metrics, including accuracy per million parameters, inference efficiency, training efficiency, and stability indices. Model robustness was then analyzed by testing the models using partially heterogeneous datasets unseen during training. Furthermore, computational complexity was compared across models to assess time and resource requirements. Explainability analyses using Grad-CAM++ and SHAP were performed to assess model interpretability and clinical relevance. Finally, the best-performing LGGC-Net model was compared with existing methods to validate its effectiveness. Together, these experiments provide a holistic assessment of both predictive performance and clinical deployment readiness.

### Binary classification

**Fig. 3:**
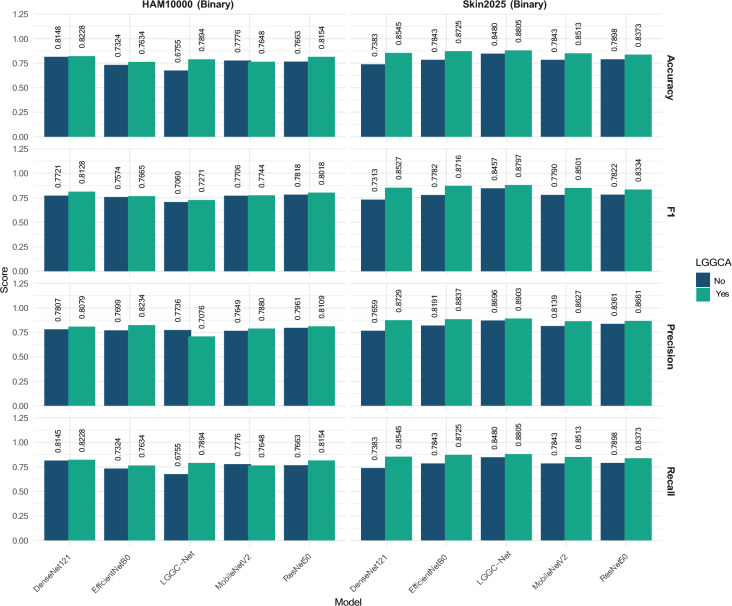
Comparison of models with/without LGGC attention based on standard metrics for binary classification (Skin2025 and HAM10000) using fixed seed.

Figure 3 shows the binary skin cancer classification performance of MobileNetV2, EfficientNetB0, DenseNet121, ResNet50 and the proposed LGGC-Net with and without the LGGC attention module. All models were trained, validated, and tested on the SKIN2025 dataset and further evaluated on the external HAM10000 dataset^3^, which was unseen during the training and validation. Across all architectures, integrating the attention module consistently improves precision, recall, F1-score, and accuracy, confirming its effectiveness in enhancing discriminative feature learning for benign–malignant classification. Among the baseline CNNs, DenseNet121 and EfficientNetB0 benefit the most from the attention integration, exhibiting notable gains in F1-score and accuracy on both datasets. This suggests that LGGC attention effectively complements dense, compound-scale feature representations by emphasizing clinically relevant lesion regions. MobileNetV2 and ResNet50 also show consistent, though comparatively smaller, performance improvements, indicating that lightweight and residual architectures gain moderate benefits from attention-guided feature refinement.

The proposed LGGC-Net achieves the highest overall performance among the evaluated models, and incorporating LGGC attention further amplifies this advantage. In particular, LGGC-Net demonstrates the most significant improvements in recall and F1-score on the external HAM10000 dataset, highlighting its superior robustness and generalization capability under heterogeneous data conditions. Overall, these results confirm that the LGGC attention module enhances binary skin cancer classification across diverse CNN architectures, with the most pronounced impact observed in deeper and attention-aware models while still providing consistent gains for lightweight networks.

**Fig. 4:**
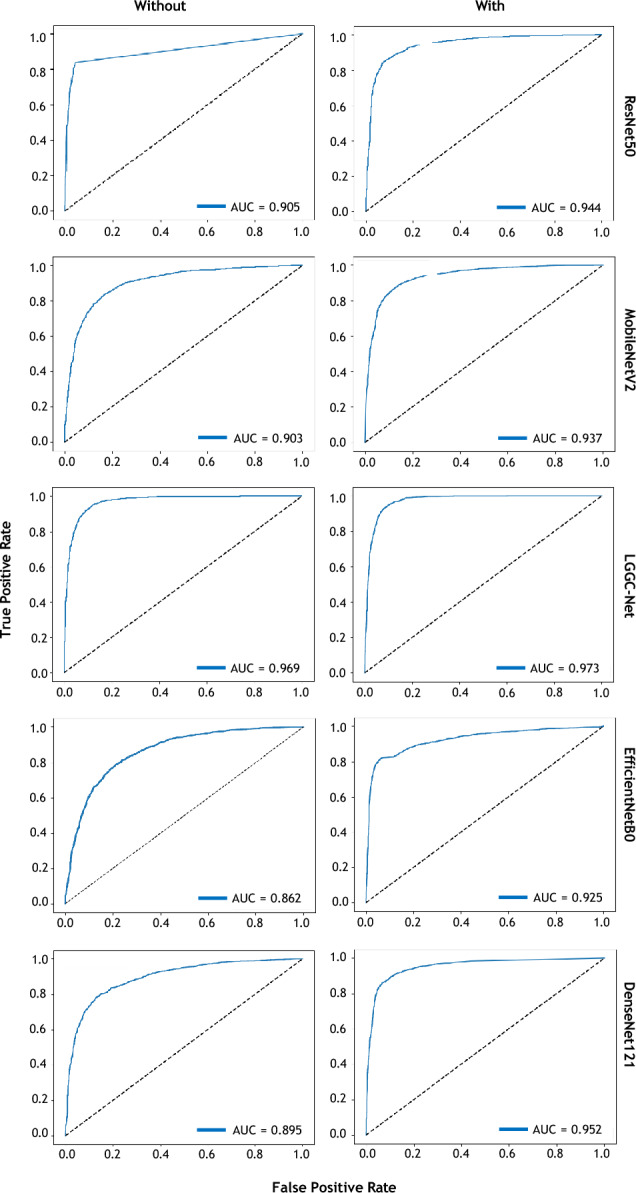
ROC curves of the models with and without LGGC attention module for binary classification (SKIN2025) using fixed seed.

The ROC–AUC analysis on the SKIN2025 test set reinforces the role of LGGC attention in improving class separability across models, as shown in Fig. 4. Without attention, baseline architectures show moderate discrimination, with lower AUC values, particularly for EfficientNetB0 (0.862) and DenseNet121 (0.895). Incorporating LGGC attention consistently increases AUC for all models, indicating more reliable sensitivity–specificity trade-offs. The most significant gains are observed for EfficientNetB0 and DenseNet121, whose AUC values rise to 0.925 and 0.952, respectively, suggesting that attention effectively enhances feature prioritization in deeper and densely connected networks. MobileNetV2 and ResNet50 also benefit, achieving AUCs of 0.937 and 0.944, respectively. The proposed LGGC-Net achieves the highest overall discrimination performance, with AUC values of 0.969 without attention and 0.973 with attention, demonstrating both strong intrinsic design and added robustness from LGGC attention. Collectively, these results confirm that LGGC attention improves threshold-independent binary classification performance on SKIN2025.

### Multiclass classification

**Fig. 5:**
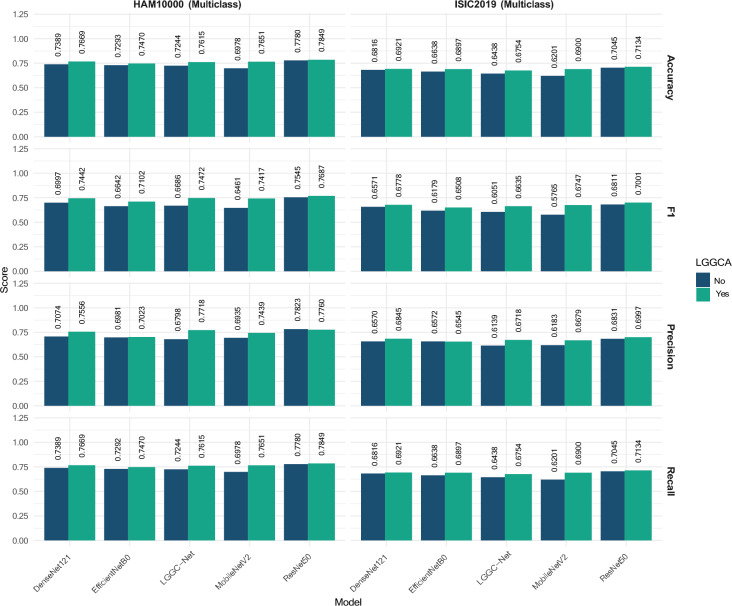
Comparison of models with/without LGGC attention based on standard metrics for multiclass classification (ISIC2019 and HAM10000) using fixed seed.

We further evaluated the effectiveness of the LGGC attention module for multi-class skin cancer classification using seven lesion subtypes from the ISIC 2019 dataset. All models were trained and validated on ISIC 2019 and subsequently tested on both ISIC 2019 and the heterogeneous HAM10000 dataset, considering only the seven overlapping classes. As summarized in Fig.  5, integrating LGGC consistently improves performance across all architectures and evaluation metrics. On the ISIC 2019 test set, DenseNet121 shows an increase in F1-score from 0.6571 to 0.6778, while MobileNetV2 improves from 0.5765 to 0.6747, indicating a substantial gain in class discrimination. Similar trends are observed on HAM10000, where MobileNetV2’s F1-score increases from 0.6461 to 0.7417, demonstrating improved generalization to unseen data. ResNet50 exhibits more modest but stable gains, with the F1-score improving from 0.6811 to 0.7001 on ISIC 2019.

The proposed LGGCNet achieves the most balanced improvement, with its F1-score increasing from 0.6051 to 0.6635 on ISIC 2019 and from 0.6686 to 0.7472 on HAM10000 when LGGC attention is applied. These results indicate that LGGC attention effectively enhances feature discrimination among visually similar lesion subtypes and improves robustness under domain shift. Overall, the consistent numerical gains across datasets confirm that LGGC attention improves multi-class classification performance and supports the development of practical, deployment-ready models for clinical use.

**Fig. 6:**
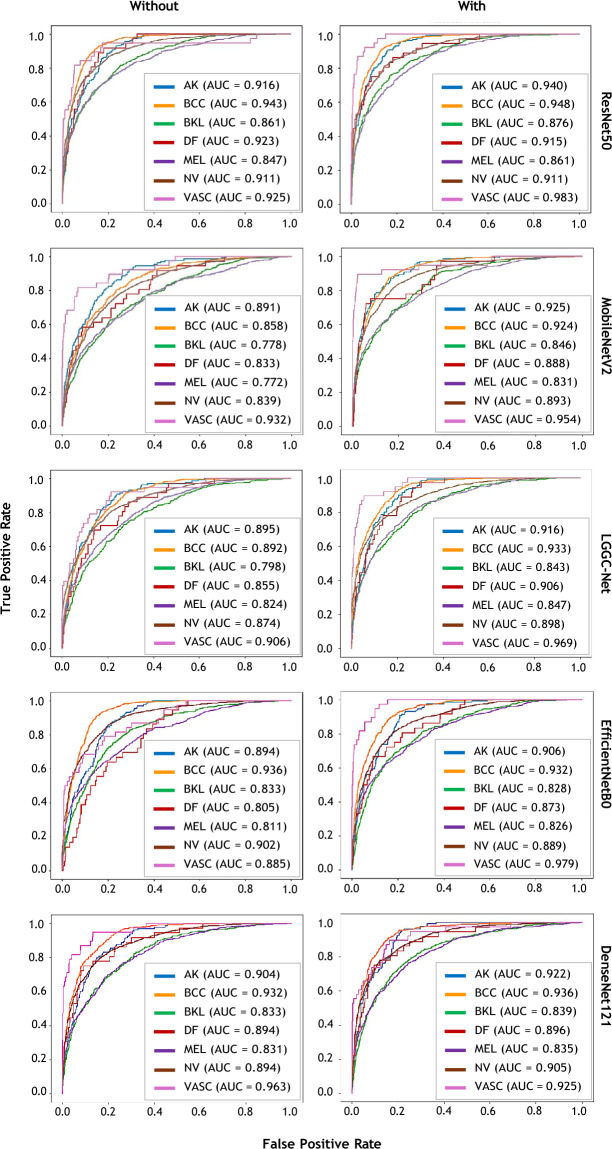
ROC curves of the models with and without LGGC attention module for multiclass classification (ISIC 2019) using fixed seed.

The class-wise AUC analysis on the ISIC 2019 dataset further highlights the contribution of the LGGC attention module in multi-class skin cancer classification. As summarized in Fig. 6, integrating LGGC attention improves or preserves AUC values across most lesion categories and model architectures, with remarkably consistent gains observed for clinically challenging classes such as BCC, BKL, MEL and NV. For instance, MobileNetV2 exhibits notable improvements in AUC for BCC (from 0.858 to 0.924), BKL (from 0.778 to 0.846) and MEL (from 0.772 to 0.831), indicating that LGGC attention substantially enhances class separability in lightweight architectures. Similarly, the proposed LGGCNet demonstrates clear gains for BKL (from 0.798 to 0.843), MEL (from 0.824 to 0.847) and VASC (from 0.906 to 0.969), reinforcing its robustness across both majority and minority lesion categories.

In terms of average AUC, LGGC attention consistently improves overall performance for ResNet50 (from 0.9037 to 0.9191), MobileNetV2 (from 0.8433 to 0.8944) and LGGCNet (from 0.8634 to 0.9017), while maintaining competitive performance for DenseNet121. Although minor class-specific fluctuations are observed for specific categories (e.g., DF or VASC in EfficientNetB0), the overall trend demonstrates that LGGC attention enhances inter-class discrimination by emphasizing salient lesion characteristics while suppressing background interference. These findings align with earlier performance analyses and confirm that the LGGC attention module strengthens multi-class decision boundaries, particularly benefiting lightweight and deployment-oriented architectures without compromising stability across dominant and minority cancer subtypes.

### Ablation study

In this study, we performed ablation experiments in two directions: (1) to evaluate the contribution of different attention mechanisms within the LGGC attention module and (2) to examine the overall impact of the LGGC attention module on model performance. Here, we report the results of analyzing the role of individual attention mechanisms. For this purpose, we applied the LGGC-Net model without the LGGC attention module on the Skin2025 balanced dataset for binary classification. Subsequently, different attention mechanisms were incorporated independently and in various combinations to investigate their effects on LGGC-Net’s performance. Table 3 summarizes the results of different attention configurations. The LGGC-Net model without any attention achieved 81.93% accuracy, serving as the baseline. When using a single attention mechanism, the color attention achieved the best performance (83.78%), highlighting its ability to capture color differences between normal and malignant lesions. The local attention provided a modest improvement (81.28%) by learning fine textures and edges. In contrast, the channel and graph attentions alone performed poorly (76.38% and 68.65%, respectively) as they emphasize global features while missing important local details.

Combining two attention mechanisms showed further improvement. The local + color pair achieved the highest accuracy (87.70%), effectively capturing both texture and color information. The color + channel combination also performed well (86.20%). In contrast, combinations including graph attention alone (e.g., local + graph or channel + graph) resulted in lower accuracy due to the loss of some fine-grained information. Incorporating three attentions together produced more stable results, with configurations such as local + color + graph and channel + color + graph reaching 85.38% and 85.54% accuracy, respectively. Finally, the complete LGGC attention module, combining all four attentions, achieved the best performance (88.05% accuracy) and the highest F1 Scores. This is because each attention contributes complementary information: local attention captures fine details, color attention emphasizes color patterns, channel attention highlights important global features, and graph attention models spatial relationships between regions. This ablation experiments confirm that each component of the LGGC attention module contributes uniquely to model performance. In particular, color and local attentions are critical for distinguishing lesion-specific features, while combining all four attentions in LGGC yields the most reliable and accurate predictions. The impact of the LGGC attention module on overall model performance is further illustrated in Figs. 3 and 5.

To ensure that the observed performance improvements are attributable to the proposed LGGC attention mechanism rather than to pretrained backbones or training configurations, we conducted a controlled evaluation across multiple CNN architectures. We evaluated four configurations for each backbone: (1) training from scratch without LGGC attention, (2) training from scratch with LGGC attention, (3) pretrained backbone without LGGC attention, and (4) pretrained backbone with LGGC attention. All models were trained using identical training protocols, datasets, and hyperparameters, and the results were averaged across multiple random seeds to ensure robustness. Table 4 presents the results along with efficiency metrics including parameter count, computational complexity (GFLOPs and GMACs), latency, throughput, GPU memory consumption, and inference time. The results demonstrate that the LGGC attention module consistently improves classification accuracy across all architectures, regardless of whether pretrained weights are used. For example, without pretraining, LGGC attention improves accuracy from 0.840 to 0.859 for DenseNet121 and from 0.746 to 0.805 for MobileNetV2. Similar improvements are observed for EfficientNetB0 and ResNet50. When combined with pretrained backbones, LGGC attention further enhances performance. Furthermore, the proposed LGGC-Net achieves the highest accuracy ($0.917 \pm 0.019$) while maintaining a significantly lower parameter count (0.812M) than standard architectures. These results confirm that the performance gains are primarily driven by the proposed attention mechanism rather than by pretrained backbones, while also maintaining a favorable efficiency-performance trade-off.

**Fig. 7:**
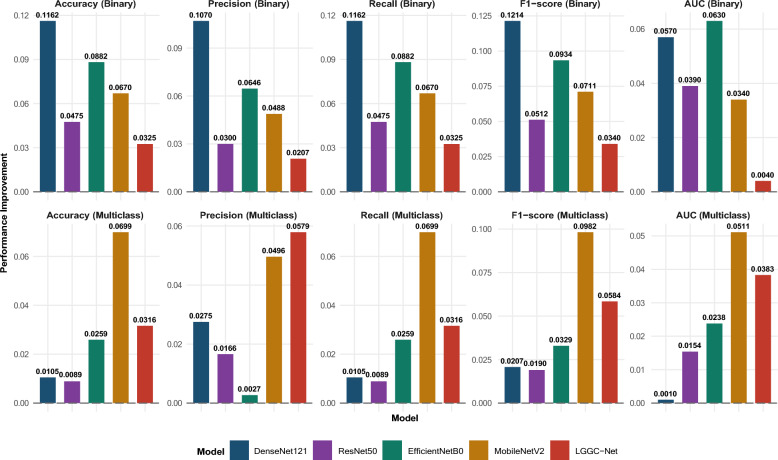
Relative performance gains achieved by LGGC attention across evaluation metrics for the models using fixed seed.

Further, Fig.  7 quantitatively demonstrates that integrating the LGGC attention module consistently improves performance across the baseline architectures and all evaluation metrics, validating its effectiveness and general applicability. For multiclass classification, LGGC yields measurable AUC gains, particularly for MobileNetV2 (+0.051) and EfficientNetB0 (+0.024), indicating improved class separability, while LGGC-Net itself shows a notable AUC improvement of +0.038. Accuracy improvements further confirm this trend, with MobileNetV2 achieving the most significant gain (+0.070) and LGGC-Net improving by +0.032. Precision and F1-score (multiclass) also increase across models, with F1-score gains reaching +0.098 for MobileNetV2 and +0.058 for LGGC-Net, reflecting a better balance between precision and recall. For binary classification, LGGC integration results in consistent gains across all backbones, with F1-score improvements ranging from +0.034 (LGGCNet) to +0.121 (DenseNet121) and accuracy gains of up to +0.116.

These results demonstrate that incorporating LGGC attention consistently improves the performance of baseline deep learning models in both binary and multiclass classification tasks. Overall, the uniform improvements across diverse architectures and metrics confirm that the LGGC attention module enhances feature representation and generalization in a model-agnostic manner. Despite achieving superior performance, LGGC-Net requires 10–55$\times$ fewer parameters than the CNN backbones such as ResNet50 and DenseNet121, highlighting its suitability for lightweight medical image analysis applications.

**Table 3:** Performance of LGGC-Net backbone with different attention variants on SKIN2025 dataset (CBAM = Convolutional Block Attention Module).

Attention variant	Avg precision	Avg recall	Avg F1-score	Accuracy
No attention	0.8378	0.8193	0.8167	0.8193
Standard Attention (CBAM)	0.8582	0.8565	0.8563	0.8565
Local	0.8314	0.8128	0.8100	0.8128
Color	0.8467	0.8378	0.8367	0.8378
Channel	0.8079	0.7638	0.7550	0.7638
Graph	0.7150	0.6865	0.6758	0.6865
Local + Color	0.8836	0.8770	0.8785	0.8770
Local + Channel	0.8477	0.8475	0.8474	0.8475
Local + Graph	0.8225	0.8110	0.8093	0.8110
Color + Channel	0.8638	0.8620	0.8618	0.8620
Color + Graph	0.8390	0.8220	0.8197	0.8220
Channel + Graph	0.8192	0.7645	0.7540	0.7645
Local + Color + Channel	0.8476	0.8473	0.8472	0.8473
Local + Channel + Graph	0.8590	0.8518	0.8510	0.8518
Local + Color + Graph	0.8581	0.8538	0.8533	0.8538
Channel + Color + Graph	0.8553	0.8554	0.8552	0.8554
Local + Channel + Graph + Color (LGGC)	0.8903	0.8805	0.8797	0.8805

**Table 4:** Controlled evaluation of LGGC attention and pretraining across the backbones using identical training protocols and multiple random seeds.

Model	Pretrained	LGGC	Accuracy	Params (M)	GFLOPs	GMACs	Latency (ms)	Throughput (img/s)	Peak GPU Mem (MB)	Inference Time (s)
DenseNet121	No	No	$0.840\pm 0.063$	7.04	5.66	2.83	92.70	10.79	191.71	514.61
	No	Yes	$\mathbf {0.859\pm 0.032}$	$\mathbf {12.42}$	$\mathbf {5.77}$	$\mathbf {2.88}$	$\mathbf {108.12}$	$\mathbf {9.25}$	$\mathbf {311.39}$	$\mathbf {577.73}$
	Yes	No	$\mathbf {0.857\pm 0.035}$	$\mathbf {7.04}$	$\mathbf {5.66}$	$\mathbf {2.83}$	$\mathbf {101.76}$	$\mathbf {9.86}$	$\mathbf {188.11}$	$\mathbf {581.37}$
	Yes	Yes	$0.903\pm 0.011$	12.42	5.77	2.88	97.34	10.33	313.96	538.38
EfficientNetB0	No	No	$0.765\pm 0.023$	4.05	0.769	0.385	79.57	12.57	114.74	475.88
	No	Yes	$\mathbf {0.790\pm 0.083}$	$\mathbf {12.47}$	$\mathbf {0.945}$	$\mathbf {0.472}$	$\mathbf {88.03}$	$\mathbf {11.44}$	$\mathbf {312.59}$	$\mathbf {512.72}$
	Yes	No	$\mathbf {0.789\pm 0.023}$	$\mathbf {4.05}$	$\mathbf {0.769}$	$\mathbf {0.385}$	$\mathbf {82.48}$	$\mathbf {12.13}$	$\mathbf {113.29}$	$\mathbf {479.17}$
	Yes	Yes	$0.813\pm 0.016$	12.47	0.945	0.472	83.67	11.95	314.62	482.32
MobileNetV2	No	No	$0.746\pm 0.031$	2.26	0.599	0.299	76.61	13.05	69.61	452.87
	No	Yes	$\mathbf {0.805\pm 0.033}$	$\mathbf {10.68}$	$\mathbf {0.774}$	$\mathbf {0.387}$	$\mathbf {83.38}$	$\mathbf {11.99}$	$\mathbf {271.88}$	$\mathbf {479.61}$
	Yes	No	$\mathbf {0.776\pm 0.019}$	$\mathbf {2.26}$	$\mathbf {0.599}$	$\mathbf {0.299}$	$\mathbf {81.02}$	$\mathbf {12.34}$	$\mathbf {73.81}$	$\mathbf {476.43}$
	Yes	Yes	$0.841\pm 0.030$	10.68	0.774	0.387	78.62	12.72	282.48	464.27
ResNet50	No	No	$0.823\pm 0.038$	23.59	7.71	3.86	91.96	10.89	629.85	502.70
	No	Yes	$\mathbf {0.835\pm 0.042}$	$\mathbf {45.12}$	$\mathbf {8.16}$	$\mathbf {4.08}$	$\mathbf {109.62}$	$\mathbf {9.13}$	$\mathbf {1138.01}$	$\mathbf {620.90}$
	Yes	No	$\mathbf {0.824\pm 0.060}$	$\mathbf {23.59}$	$\mathbf {7.71}$	$\mathbf {3.86}$	$\mathbf {92.01}$	$\mathbf {10.87}$	$\mathbf {647.36}$	$\mathbf {546.19}$
	Yes	Yes	$0.873\pm 0.065$	45.12	8.16	4.08	91.82	10.89	1122.90	517.70
LGGC-Net	No	No	$0.857\pm 0.017$	0.358	0.349	0.174	81.07	12.34	24.53	486.80
	No	Yes	$\mathbf {0.917\pm 0.019}$	$\mathbf {0.812}$	$\mathbf {2.33}$	$\mathbf {1.17}$	$\mathbf {81.43}$	$\mathbf {12.28}$	$\mathbf {111.91}$	$\mathbf {474.39}$

### Deployment compatibility

**Fig. 8:**
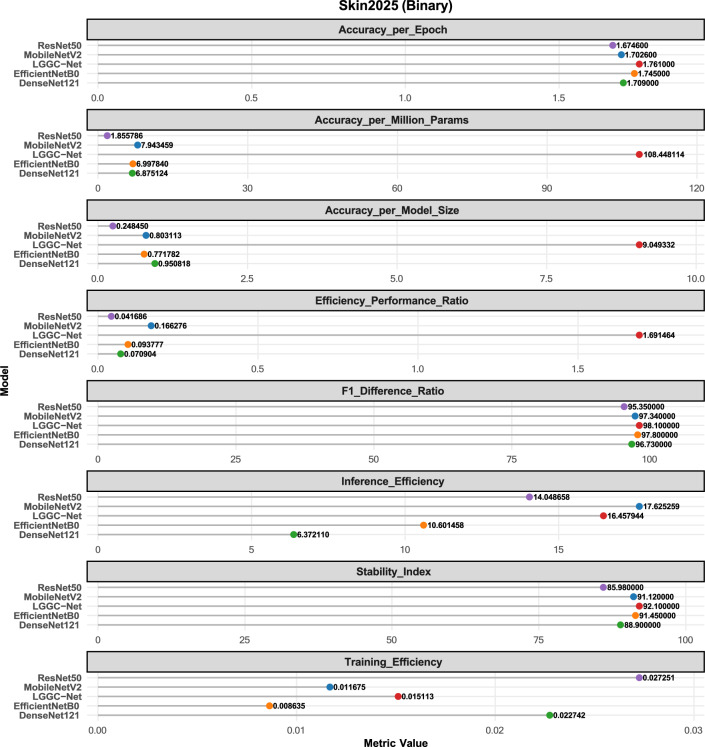
Comparison of models equipped with LGGC attention based on deployment-oriented metrics for binary classification (SKIN2025) using fixed seed.

Figure 8 shows the comparison of the five models on the SKIN2025 dataset with respect to different metrics of efficiency and performance. With respect to Accuracy per Million Parameters, LGGC-Net has a large margin with a value of 1.084481, which is significantly higher than other models (MobileNetV2: 0.079435, EfficientNetB0: 0.069978, DenseNet121: 0.068751, ResNet50: 0.018558), showing that LGGC-Net has the highest accuracy in terms of the minimum number of parameters. LGGC-Net is also the best performer in terms of Accuracy per Model Size (0.090493) and Efficiency–Performance Ratio (0.016915), demonstrating that it provides better accuracy with minimal size and cost. Regarding the F1 Difference Ratio, which measures the difference between malignant and benign classifications, ResNet50 has the highest value (0.953500), although all models have relatively similar measures. Regarding inference efficiency, MobileNetV2 has the highest value (0.176253) among the models, indicating faster inference. However, DenseNet121 has the lowest measure (0.063721) compared with other models. Regarding the Stability Index, MobileNetV2 has the highest value (0.911200) among the models, indicating better sensitivity and specificity. Regarding Training Efficiency, DenseNet121 has the highest value (0.000227) among the models, indicating better efficiency than the others, although LGGC-Net (0.000151) and MobileNetV2 (0.000117) have higher values. The figure makes it clear that LGGC-Net is the most parameter-efficient and size-efficient model, MobileNetV2 is the fastest in inference, ResNet50 shows strong F1 consistency, and DenseNet121 trains most efficiently. Overall, the figure clearly shows that LGGC-Net is the best and most balanced model. It delivers high accuracy, runs efficiently, and remains stable, making it the most suitable model for real-world use.

**Fig. 9:**
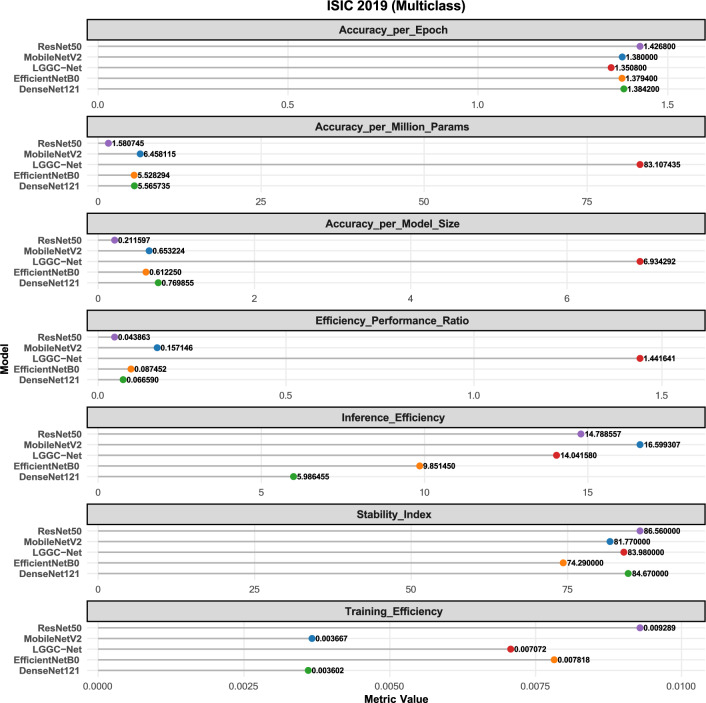
Comparison of models equipped with LGGC attention based on deployment-oriented metrics for multiclass classification (ISIC 2019) using fixed seed.

Figure 9 compares the performance and efficiency of five models on the ISIC 2019 dataset using different metrics. DenseNet121 gives moderate results, with 0.059 inference efficiency and 0.007 accuracy per model size, showing stable but slower behavior. EfficientNetB0 performs slightly better, achieving 0.098 in inference efficiency and 0.006 in accuracy per model size, making it a balanced but not top-performing model. MobileNetV2 shows strong speed-oriented performance, with 0.165 inference efficiency and 0.064 accuracy per million parameters, which makes it a good lightweight option. ResNet50 gives the highest accuracy-related values, including 0.831 accuracy per million parameters, 0.147 inference efficiency, and the best stability index of 0.865, proving it is the strongest overall performer. LGGC-Net stands out in efficiency-focused metrics, especially with the highest efficiency–performance ratio of 0.014 and an inference efficiency of 0.140, demonstrating that it provides excellent performance despite being a small model. Overall, the figure clearly highlights that ResNet50 offers the best accuracy and stability. At the same time, LGGC-Net delivers the best efficiency while maintaining competitive performance, making these two models the strongest choices depending on the requirements.

### Robustness and generalization analysis

To evaluate model robustness and generalization capability, all trained models were externally tested on the HAM10000 dataset. Although HAM10000 originates from the same ISIC archive^56^, care was taken to ensure that all images used for external evaluation were completely excluded from the training and validation sets. Consequently, HAM10000 serves as a partially heterogeneous dataset, introducing variations in acquisition conditions, class distribution, and patient samples. For binary classification, models were trained on the SKIN2025 dataset; for multiclass classification, they were trained on the ISIC 2019 dataset. This external evaluation assessed the stability of learned representations under domain shift and heterogeneous data conditions. Further, we tested the binary classifier on a entirely heterogeneous dataset DST-50.

**Fig. 10:**
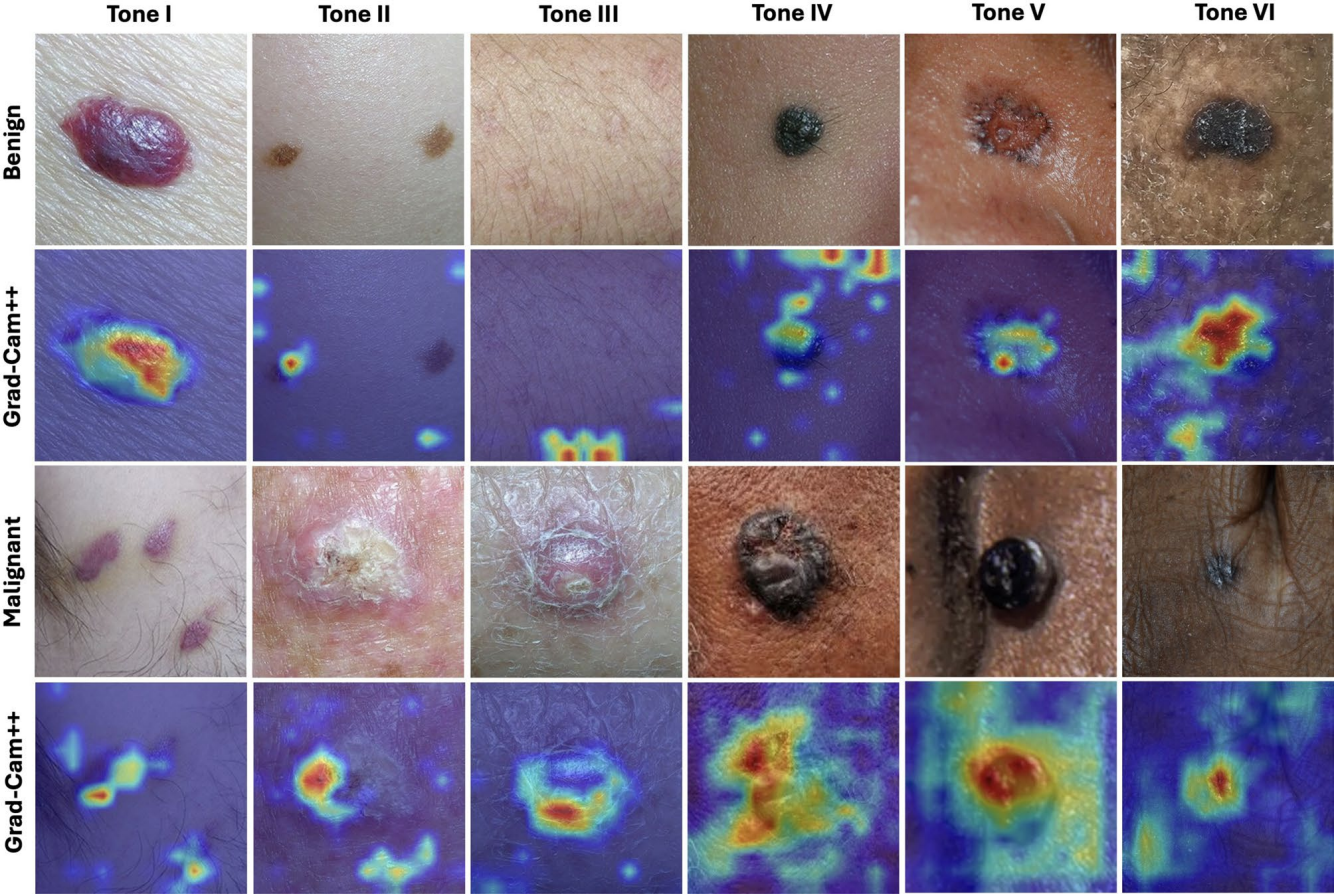
LGGC-Net based Grad-CAM++ on images spanning the entire Fitzpatrick skin tone scale (I–VI).

For binary classification, the results indicate that integrating the LGGC attention module generally improves robustness across different architectures. DenseNet121 with LGGC attention achieved the strongest and most balanced performance on HAM10000, reaching an F1-score of 0.8128 and an accuracy of 0.8228, compared to 0.7721 and 0.8145 without attention. ResNet50 also exhibited notable robustness gains, with its F1 Score improving from 0.7818 to 0.8018 and its accuracy increasing from 0.7663 to 0.8154. EfficientNetB0 benefited from attention primarily through enhanced precision (from 0.7699 to 0.8234) and overall accuracy (from 0.7324 to 0.7634). Although MobileNetV2 showed mixed behavior, its F1-score remained stable with attention (from 0.7706 to 0.7744), indicating consistent generalization. The proposed LGGC-Net demonstrated a clear robustness improvement, with recall increasing substantially from 0.6755 to 0.7894, highlighting the attention module’s effectiveness in reducing false negatives under distribution shift. On the DST-50 dataset, LGGC-Net achieved an accuracy of 85%, correctly classifying 43 benign and 42 malignant images. This performance indicates that the model is unbiased and highly adaptable, consistently predicting lesions across diverse skin tones. Furthermore, we demonstrate the generalization ability of LGGC-Net through Grad-CAM++ visualizations on images spanning the entire Fitzpatrick skin tone scale (I–VI), as shown in Fig.  10.

For multiclass classification, LGGC attention consistently enhanced external performance on the HAM10000 dataset. DenseNet121 improved its average F1-score from 0.6997 to 0.7442, while MobileNetV2 showed a marked increase from 0.6461 to 0.7417, reflecting improved discrimination across multiple lesion subtypes. LGGC-Net again demonstrated strong robustness gains, with its average F1-score increasing from 0.6686 to 0.7472 and accuracy improving from 0.7244 to 0.7615. These improvements were achieved despite differences in class distributions between the ISIC 2019 and HAM10000 datasets.

Overall, the external evaluation confirms that the LGGC attention module enhances robustness by guiding models toward clinically relevant and transferable features, thereby reducing sensitivity to dataset-specific variations. The observed improvements in recall and F1-score are particularly important for reliable deployment in real-world clinical environments, where data heterogeneity is unavoidable.

### Computational complexity

**Fig. 11:**
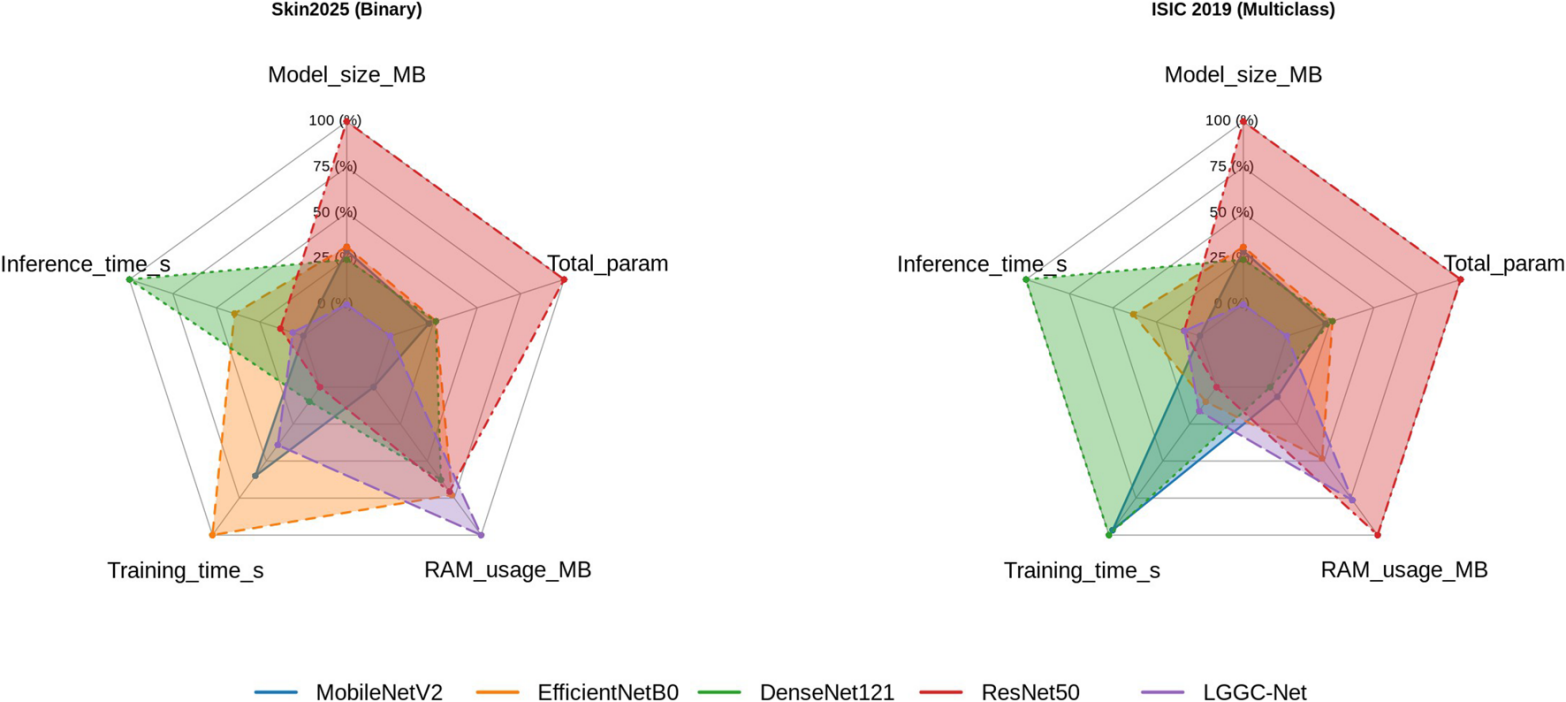
Efficiency differences among models: MobileNetV2, EfficientNetB0, DenseNet121, ResNet50 and LGGC-Net using fixed seed.

Figure 11 presents two radar plots comparing the computational efficiency of five models: MobileNetV2 (blue), EfficientNetB0 (orange), DenseNet121 (green), ResNet50 (red), and the proposed LGGC-Net (purple) across five efficiency metrics for two datasets. The left radar plot corresponds to the binary-class SKIN2025 dataset, while the right plot corresponds to the multiclass ISIC 2019 dataset. In the Skin2025 dataset, LGGC-Net clearly stands out as the most lightweight model, with the smallest model size (9.73 MB) and the fewest parameters (811,909). By comparison, MobileNetV2 (106 MB, 10.7M parameters), EfficientNetB0 (113.05 MB, 12.46M parameters), DenseNet121 (89.87 MB, 12.42M parameters) and ResNet50 (337.01 MB, 45.1M parameters) are considerably larger. Regarding inference time, DenseNet121 is the slowest (13.41 s), whereas MobileNetV2 (4.83 s), LGGC-Net (5.35 s), and ResNet50 (5.96 s) are faster. Training time varies across models, with EfficientNetB0 requiring the longest (10,104.5 s) and ResNet50 the fastest (3,072.52 s). In terms of RAM usage, LGGC-Net consumes the most memory (1,641.95 MB), followed by EfficientNetB0 and ResNet50. The ISIC2019 multiclass dataset shows a similar trend. LGGC-Net remains the smallest model (9.74 MB, 812,683 parameters), while ResNet50 remains the largest (337.15 MB, 45.13M parameters). DenseNet121 again has the slowest inference time (11.56 s), whereas MobileNetV2 (4.16 s), LGGC-Net (4.81 s), and ResNet50 (4.82 s) perform faster. DenseNet121 also requires the longest training time (19,212.62 s), with ResNet50 training fastest (7,680.12 s). RAM usage increases for all models on this dataset, with ResNet50 using the most (2,131.87 MB) and LGGC-Net also using a high amount (2,024.07 MB).

Overall, these radar plots highlight clear trade-offs among the models. ResNet50 is the heaviest and most memory-intensive model; DenseNet121 is consistently the slowest; MobileNetV2 offers stable, efficient performance; and EfficientNetB0 is moderate but resource-demanding. LGGC-Net emerges as the most lightweight architecture, with small size and minimal parameters, while still achieving competitive inference speed for both binary and multiclass classification tasks.

### Explainability analysis

**Fig. 12:**
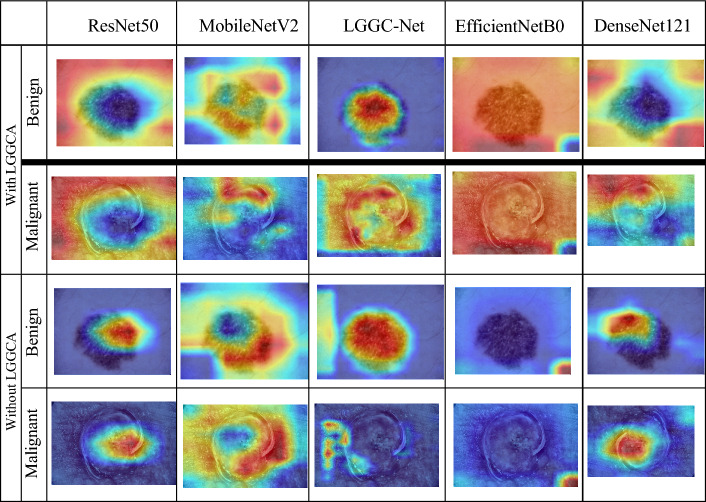
Grad-Cam++ visualization.

To interpret the decision-making ability of the models, we conducted an explainability analysis using both Grad-CAM++ and SHAP visualizations. Figure 12 shows the Grad-CAM++ visualizations of the models. Grad-CAM++ identifies which regions of skin lesion images contribute most to the model’s predictions by computing the first, second, and third-order gradients of the model’s output with respect to the last convolutional layer’s feature maps. The importance weights for each spatial location are calculated as:

$$\alpha _{ij}^k = \frac{ \frac{\partial ^2 y^c}{\partial (A_{ij}^k)^2} }{ 2\left( \frac{\partial ^2 y^c}{\partial (A_{ij}^k)^2}\right) + \left( \frac{\partial ^3 y^c}{\partial (A_{ij}^k)^3}\right) A_{ij}^k + \epsilon }.$$

The final heatmap is obtained by combining these weights with the positive gradients:

$$L_{\text {Grad-CAM++}}^c = \text {ReLU}\left( \sum _k \left( \sum _{i,j} \alpha _{ij}^k \cdot \max \left( \frac{\partial y^c}{\partial A_{ij}^k}, 0 \right) \right) A^k \right) .$$

After normalization and resizing, the heatmap is overlaid on the original image to highlight the regions that influenced the model’s decision.

**Fig. 13:**
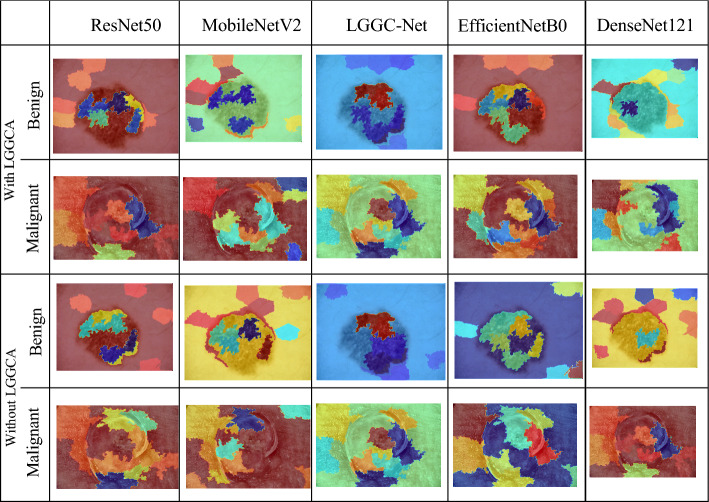
SHAP visualization.

The results demonstrate that integrating the LGGC attention module substantially improves model explainability. Models with LGGC attention focus more precisely on lesion-specific regions, producing more precise and more localized activation maps that align with the actual structures of benign and malignant patterns. In contrast, models without LGGC attention show scattered activations and often highlight irrelevant areas. Among the evaluated architectures, LGGC-Net with LGGC attention achieves the best explainability, with activation maps highly concentrated on lesion regions, making predictions more interpretable and relatable for dermatologists. We further applied SHAP to identify which regions of each skin lesion image contributed most to model predictions. Figure 13 presents the SHAP superpixel visualizations. Each image was segmented into approximately 50 regions using the SLIC algorithm, and SHAP evaluated how perturbing each region affected the model’s output. The resulting values were visualized as a heatmap using the JET color map, where red and yellow indicate strong support for the prediction, blue indicates opposition, and green or light-blue indicates minimal impact. The heatmap was overlaid on the original image to highlight regions influencing the model’s decision. Comparison across models with and without LGGC attention shows that LGGC attention consistently improves explainability, similar to Grad-CAM++ results. Models with LGGC attention focus clearly on lesion regions, while models without it often attend to irrelevant background areas. LGGC-Net with LGGC produces the most precise and clinically meaningful SHAP maps, concentrating on lesion-specific regions that align with dermatologists’ expectations.

Overall, the combination of Grad-CAM++ and SHAP analyses confirms that the LGGC attention module guides the models to focus on clinically relevant regions. Grad-CAM++ highlights the spatial attention in convolutional layers, while SHAP quantifies the contribution of superpixel regions to the prediction. Together, these complementary methods ensure that our models, particularly LGGC-Net, provide interpretable and trustworthy predictions that are aligned with clinical reasoning. From a clinical perspective, these explainability outputs can support the integration of AI systems into dermatological workflows. The Grad-CAM++ visualizations allow clinicians to verify whether the model focuses on clinically relevant lesion regions, while SHAP provides complementary insights into which specific image regions contribute most to the prediction. Such explanations can assist dermatologists in validating model outputs, identifying potential model errors, and improving confidence in AI-assisted screening tools. Therefore, the proposed framework is intended to support clinical screening and decision-making rather than providing a definitive diagnosis.

### Comparative study

Table 5 presents a comprehensive comparison between the proposed LGGC attention-based models and existing skin cancer image classification methods. In addition to conventional performance metrics such as accuracy, F1-score, and AUC, this study emphasizes deployment-oriented evaluation metrics, such as accuracy per epoch and accuracy per million parameters. These metrics directly reflect training efficiency and parameter efficiency, which are critical considerations for real-world clinical deployment where computational resources, training time, and scalability are often constrained. We used the same number of epochs as the original model to compute accuracy-per-epoch metrics for datasets evaluated only during testing, such as the DST-50 and HAM10000 test sets. It should be noted that the values reported in Table 5 are compiled from the corresponding original publications; therefore, differences in experimental settings may influence the reported results, and the comparison should be interpreted as indicative rather than a strict head-to-head evaluation.

From a conventional performance standpoint, several existing methods report higher absolute accuracy or F1 Scores, particularly those that rely on large-scale architectures such as Swin Transformers, Vision Transformers (ViT), VGG, DenseNet, and EfficientNet variants, trained for extended schedules of 100-400 epochs. However, such models typically contain tens to hundreds of millions of parameters and incur high computational and training costs. In contrast, the proposed LGGC-Net is trained for only 50 epochs and contains approximately 0.81 million parameters, thereby naturally limiting peak accuracy but significantly improving training and deployment efficiency. When evaluated using the proposed deployment-oriented metrics, LGGC-Net demonstrates clear advantages over existing methods. Specifically, LGGC-Net achieves substantially higher accuracy per epoch across multiple datasets, including 1.761 on SKIN2025 (binary), 1.579 on HAM10000 (binary), 1.351 on ISIC 2019 (multi-class), and 1.523 on HAM10000 (multi-class). These values are consistently higher than those of large-scale models trained for longer durations, indicating faster convergence and superior training efficiency. This characteristic is particularly valuable in clinical settings where rapid model adaptation and retraining may be required.

Moreover, LGGC-Net exhibits exceptionally high accuracy per million parameters, achieving values of 108.7 on SKIN2025 (binary), 97.46 on HAM10000 (binary), 83.38 on ISIC 2019 (multi-class), and 94.01 on HAM10000 (multi-class). These results significantly surpass those of existing CNN- and transformer-based methods, including compact models such as MobileNet and NASNet, as well as their lightweight variants. The high accuracy per million parameter values highlights the strong discriminative capability of the proposed LGGC attention mechanism despite its minimal parameter footprint.

In addition to efficiency advantages, LGGC-Net consistently achieves high AUC values across datasets, including 0.973 on SKIN2025 (binary), 0.901 on ISIC 2019 (multi-class), and 0.931 on HAM10000 (multi-class), demonstrating robust class separation even under the class imbalance conditions commonly observed in dermatological datasets. This behavior is particularly relevant for clinical screening applications, where reliable discrimination between malignant and benign lesions is often more critical than marginal gains in overall accuracy. Although the proposed LGGC attention-based models may not always outperform state-of-the-art methods in absolute accuracy, they clearly excel when evaluated using deployment-oriented metrics, such as accuracy per epoch and accuracy per million parameters. These results confirm that LGGC-Net offers a superior balance between performance, efficiency, and practicality, making it a strong candidate for scalable, cost-effective, and accessible skin cancer classification in real-world clinical environments.

**Table 5:** Comparison of proposed LGGC attention based models with existing methods. Reported values are taken from original publications to provide a reference-level comparison; differences in experimental settings may affect the results. (E=Epochs, P= Parameters, A= Accuracy, Pr=Precision, R=Recall, F1=F1-score, AUC= Area under the curve value, AE=Accuracy per epoch, AP=Accuracy per million parameters, M=million and – = Not available).

Ref.	Model	Dataset	A	Pr	R	F1	AUC	AE	AP
^22^	**SKINC-NET (E:100 , P:0.66M )**	HAM10000 (multi)	**0.984**	**0.970**	**0.980**	**0.990**	–	**0.984**	**146.9**
^14^	TurkerNet (E:50 , P:10M )	Kaggle (binary)	0.921	0.925	0.930	0.919	–	1.842	9.210
^15^	EfficientNetB4 (E:200 , P:18M)	HAM10000 (multi)	0.858	–	0.697	–	0.869	0.429	4.888
^16^	LViT (E:100 , P:86M)	ISIC 2023 (binary)	0.958	0.936	0.867	0.900	–	0.958	1.113
^18^	SkinSwinViT (E:100 , P:31M)	ISIC 2018 (multi)	0.978	0.975	0.978	0.977	–	0.978	3.154
^17^	HI-MViT (E:50 , P:4.5M)	ISIC 2018 (multi)	0.932	0.931	0.932	0.931	0.977	1.864	20.71
^19^	TinyStudent (E:120 , P:1.05M)	HAM10000 (multi)	0.880	–	–	0.880	–	0.733	83.80
^20^	DC-DenseNet121 (E:50 , P:7.48M)	HAM10000 (multi)	0.855	–	–	–	0.845	1.710	11.43
^21^	ConvNeXtV2 (E:– , P:21.9M)	ISCI 2019 (multi)	0.934	0.932	0.907	0.918	0.845	–	4.264
^25^	VGG16 (E: 400, P: 138M)	ISIC 2019 (multi)	0.798	0.798	0.699	0.745	–	0.199	0.578
	ResNet50 (E: 400, P: 25.6M)	ISIC 2019 (multi)	0.751	0.664	0.684	0.674	–	0.188	2.930
	DenseNet121(E: 400, P: 7.98M)	ISIC 2019 (multi)	0.783	0.737	0.724	0.730	–	0.196	9.820
	EfficientNetV2 (E:400, P:54M)	ISIC 2019 (multi)	0.827	0.785	0.775	0.780	–	0.207	1.530
	Swin transformer (E:400, P:88M)	ISIC 2019 (multi)	0.893	0.882	0.851	0.866	–	0.223	1.020
^26^	NASNetMobile (E:150, P:5.3M)	ISIC 2017 (multi)	0.774	0.779	–	0.751	0.868	0.516	14.61
	MobileNetV2-32 (E:150, P:3.5M)	ISIC 2017 (multi)	0.785	0.788	–	0.780	0.897	0.524	22.44
	MobileNetV2-64 (E:150, P:4.2M)	ISIC 2017 (multi)	0.776	0.762	–	0.756	0.888	0.518	18.49
^8^	ECRNet (E:100, P:54.18M)	ISIC 2018 (multi)	0.921	0.888	0.860	0.872	–	0.921	1.700
	ECRNet (E:100, P:54.18M)	XJUSK (multi)	0.862	0.839	0.815	0.820	–	0.862	1.590
^27^	ViT (E:– , P:86M)	MCVSLD (multi)	0.770	–	–	0.730	–	–	0.900
	EfficientNetB5 (E:– , P:30M)	MCVSLD (multi)	0.860	–	–	0.830	–	–	2.870
	DinoV2 Large (E:– , P:300M)	PAD-UFES(multi)	0.840	–	–	0.830	–	–	0.280
^58^	LcmUNet (E:100 , P:1.49M )	BUSI (multi)	0.766	0.766	0.799	0.773	–	0.766	51.40
	LcmUNet (E:100 , P:1.49M)	ISIC 2018 (multi)	0.929	0.929	0.920	0.918	–	0.929	62.30
^28^	ResNet (E:–, P:25.6M)	ISIC Archive (multi)	0.502	0.520	0.500	0.510	0.500	–	1.960
	VGGNet (E:– , P:138M)	ISIC Archive (multi)	0.487	0.480	0.460	0.470	0.480	–	0.352
^29^	TCC-NetV1 (E:– , P:5.6M)	ISIC 2019 (multi)	0.750	–	–	0.550	–	–	13.39
	EfficientNetV1B1 (E:– , P:6.5M)	ISIC 2019 (multi)	0.700	–	–	0.450	–	–	10.77
	DenseNet121 (E:– , P:7M)	ISIC 2019 (multi)	0.770	–	–	0.560	–	–	11.00
	EfficientNetV2S (E:– , P:20.3M)	ISIC 2019 (multi)	0.720	–	–	0.470	–	–	3.550
	XceptionNet (E:– , P:20.8M)	ISIC 2019 (multi)	0.750	–	–	0.540	–	–	3.610
	ResNet50 (E:– , P:23.6M)	ISIC 2019 (multi)	0.630	–	–	0.360	–	–	2.670
**Ours**	**LGGC-Net** (E:50, P:0.81M)	SKIN2025 (binary)	**0.880**	0.879	0.880	0.890	0.973	**1.761**	**108.7**
	EfficientNetB0 (E:50, P:12.46M)	SKIN2025 (binary)	0.872	0.883	0.872	0.871	0.925	1.745	7.000
	DenseNet121 (E:50, P:12.42M)	HAM10000 (binary)	0.822	0.807	0.822	0.812	0.807	1.646	6.630
	**LGGC-Net** (E:50, P: 0.81M)	HAM10000 (binary)	**0.789**	0.707	0.789	0.727	–	**1.579**	**97.46**
	**LGGC-Net** (E:50, P: 0.81M)	DST-50 (binary)	**0.850**	0.857	0.840	0.848	–	**1.700**	**104.9**
	ResNet50 (E:50 , P:45.13M)	ISIC 2019 (multi)	0.713	0.699	0.713	0.700	0.919	1.427	1.580
	**LGGC-Net** (E:50 , P:0.812M)	ISIC 2019 (multi)	**0.675**	0.671	0.6754	0.663	**0.901**	**1.351**	**83.38**
	ResNet50 (E:50, P:45.13M)	HAM10000 (multi)	0.784	0.776	0.785	0.768	0.940	1.570	1.740
	**LGGC-Net** (E:50 , P:0.812M)	HAM10000 (multi)	**0.761**	0.771	0.761	0.747	**0.931**	**1.523**	**94.01**

## Discussion

In this paper, we propose the LGGC attention module and systematically evaluate its effectiveness when integrated with standard CNN backbones and the proposed lightweight LGGC-Net for skin cancer classification. The experimental results validate the contributions of this study, with particular emphasis on performance improvement, robustness, efficiency, explainability, and deployment feasibility.

Across both binary and multiclass classification tasks, the results consistently demonstrate that integrating the LGGC attention module improves model performance regardless of the underlying backbone architecture. In binary classification experiments on the SKIN2025 and external HAM10000 datasets, all CNN models benefited from LGGC attention across precision, recall, F1-score, accuracy, and AUC. The most notable gains were observed in DenseNet121, EfficientNetB0, and the proposed LGGC-Net, indicating that LGGC attention effectively enhances feature discrimination by emphasizing clinically relevant lesion regions. These improvements were especially notable on the external HAM10000 dataset, highlighting the attention module’s ability to support generalization under partially heterogeneous data conditions, a critical requirement for real-world clinical deployment.

In multiclass classification on ISIC 2019 and HAM10000, LGGC attention again provided consistent benefits across all evaluated models. Improvements in F1-score and average AUC indicate enhanced inter-class discrimination, particularly among visually similar lesion subtypes such as BKL, MEL, BCC, and NV. This is clinically significant, as misclassification among these categories often leads to diagnostic uncertainty. Overall, these results confirm that LGGC attention captures complementary information related to local texture, color distribution, global channel relevance, and spatial relationships, all of which are essential cues in dermatological image analysis.

The ablation study provides direct evidence supporting the design rationale of the LGGC attention module. Individual attention components alone were insufficient to achieve optimal performance, and certain mechanisms, such as graph or channel attention in isolation, showed limited effectiveness. In contrast, their combined integration, particularly local and color attention, resulted in substantial performance gains. The complete LGGC attention module achieved the highest accuracy and F1-score, confirming that each component contributes unique and complementary information. This validates the core contribution of this study: a carefully designed multi-attention framework that effectively integrates fine-grained local features, color patterns, global channel importance and spatial relationships.

A major strength of this work is its focus on deployment-oriented evaluation. While many existing studies prioritize absolute accuracy using large models trained over long schedules, this study demonstrates that competitive performance can be achieved with significantly fewer parameters and shorter training durations. LGGC-Net, with approximately 0.81 million parameters, consistently achieves high AUC values and competitive accuracy across datasets despite being trained for only 50 epochs. Deployment-oriented metrics further indicate that LGGC-Net provides an optimal balance between accuracy, parameter efficiency, and overall computational cost, making it well-suited for resource-constrained environments such as hospitals, clinics, and edge devices. This efficiency is enabled by the design of the proposed LGGC attention module, which enhances feature representation by integrating local, color-aware, channel (global) and graph-based attention within a unified framework. Each component is structured to maintain computational efficiency: local attention employs depthwise separable convolutions with linear complexity $\mathscr {O}(HWC)$, while color-aware and channel attention introduce relatively modest overheads of $\mathscr {O}(C^2)$ and $\mathscr {O}(C^2/r)$, respectively. The graph attention module captures long-range dependencies with complexity $\mathscr {O}(N^2C)$; however, the number of nodes *N* is reduced through spatial downsampling, thereby mitigating the associated computational cost. Overall, the combination of lightweight operations and reduction strategies allows the LGGC attention module to balance representational richness with computational efficiency, as reflected in the reported FLOPs, latency, and memory usage. Nevertheless, the graph-based component remains the primary source of additional computational overhead. Consequently, in highly resource-constrained scenarios (e.g., edge devices or real-time applications), simplified or reduced variants of the attention module may be more suitable. This highlights a practical trade-off between modeling capacity and computational efficiency, enabling the architecture to be adapted according to deployment requirements.

The robustness analysis further reinforces the practical value of LGGC attention. External testing on the HAM10000 dataset revealed consistent improvements in recall and F1-score for both binary and multiclass classification. Notably, LGGC-Net exhibited substantial recall gains under domain shift, indicating a reduction in false negatives, which is especially important for skin cancer screening. These results suggest that LGGC attention encourages models to learn transferable and clinically meaningful representations rather than dataset-specific patterns. Computational complexity analysis further highlights that LGGC-Net is the most lightweight architecture while maintaining competitive inference speed. Although it consumes relatively higher RAM during execution, its tiny model size and low parameter count make it favorable for deployment where storage and model transfer constraints are critical.

Explainability analyses using Grad-CAM++ and SHAP demonstrate that models equipped with LGGC attention consistently produce more localized, clinically relevant activation maps that focus on lesion regions rather than background artifacts. This improved interpretability enhances transparency and trust, which are essential requirements for clinical decision support systems. Compared with existing methods, although some state-of-the-art models achieve higher absolute accuracy, they rely on large architectures, extensive training schedules, and high computational costs. In contrast, LGGC attention-based models, particularly LGGC-Net, achieve strong AUC and competitive accuracy with orders of magnitude fewer parameters and significantly lower training complexity, making them more practical for real-world deployment.

Despite these promising results, this study has several limitations. A major limitation is the inherent class imbalance present in dermoscopic datasets, which reflects real-world clinical prevalence but poses challenges for deep learning-based classification. Such an imbalance can bias models toward the majority class and adversely affect threshold-dependent metrics, such as accuracy, even when discriminative capability is strong. This behavior is evident in our results, where several models achieved moderate accuracy (approximately 80-90%) while maintaining consistently high AUC values (>0.90). The high AUC indicates effective class separability across varying decision thresholds, whereas fixed thresholds and skewed class distributions influence the comparatively lower accuracy. To mitigate this issue, class-sensitive metrics such as AUC, F1-score, precision, and recall were emphasized, and deployment-oriented metrics for efficiency, robustness, and inference feasibility were incorporated to balance predictive performance and clinical applicability. Another limitation of this study is the limited diversity of the datasets, despite evaluating the proposed framework on multiple datasets. The ISIC and HAM10000 datasets predominantly represent lighter skin tones (Fitzpatrick I–II) and originate from similar clinical settings, resulting in only partial domain variation. To examine adaptability across darker skin tones, we additionally evaluated the model on the DST-50 dataset (Fitzpatrick III–VI). However, due to its limited size, this analysis should be interpreted as a preliminary evaluation rather than a comprehensive assessment of fairness. Consequently, the reported results demonstrate robustness under partial domain shift but do not imply full population-level generalization.

Although the proposed LGGC-Net demonstrates consistent performance improvements across both binary and multiclass classification tasks, the absolute multiclass accuracy values should be interpreted with caution in a clinical context. While the results are comparable to or better than several existing AI-based approaches, further validation under real clinical conditions is required before considering its use for fully autonomous clinical diagnosis. Instead, the proposed framework is better positioned as a screening or decision-support tool that assists clinicians in identifying potentially suspicious lesions for further examination.

In summary, the experimental results collectively support the main contribution of this work which is the development of the LGGC attention module and its effective integration with CNN architectures, particularly the lightweight LGGC-Net. The observed improvements in performance, robustness under partial domain shift, parameter efficiency, and explainability indicate the potential of the proposed approach for practical skin lesion analysis. In addition, the consideration of deployment-oriented efficiency metrics highlights its suitability for resource-constrained environments. While further validation on larger and more diverse clinical datasets is required, the proposed framework represents a promising step toward reliable and clinically useful AI-assisted skin cancer screening systems.

## Conclusion

This study presents LGGC-Net, a lightweight CNN that integrates local–global graph and color attention to achieve accurate and efficient skin cancer classification. Extensive experiments demonstrate that LGGC-Net consistently improves diagnostic performance and generalization while maintaining low computational complexity. Its strong efficiency–accuracy trade-off, robustness under domain shift and compatibility with resource-constrained environments highlight its potential for real-world clinical deployment.

## Data Availability

The datasets used in this research is publicly available and can be accessed via these links: [ISIC Archive (SKIN2025)](https:/challenge.isic-archive.com) , [ISIC](https:/challenge2019.isic-archive.com) [2019](https:/challenge2019.isic-archive.com) and [HAM10000](https:/api.isic-archive.com/collections/66) . The code used in this study is publicly available at: [GitHub Code](https:/github.com/aminur-sarker6232/Skin-Lesion-Analysis-Using-CNNs)
